# Beneficial Effect of Platelet-Rich Fibrin as an Adjunct to Nonsurgical Therapy After Subgingival Professional Mechanical Plaque Removal for Periodontitis: A Systematic Review and Meta-Analysis

**DOI:** 10.3390/clinpract15070127

**Published:** 2025-07-02

**Authors:** Monica Tanady, Fatimah Maria Tadjoedin, Sri Lelyati C. Masulili, Nadhia Anindhita Harsas, Adityo Widaryono

**Affiliations:** 1 Undergraduate Program, Faculty of Dentistry, Universitas Indonesia, Jakarta 10430, Indonesia; monica.tanady11@ui.ac.id; 2 Department of Periodontology, Faculty of Dentistry, Universitas Indonesia, Jakarta 10430, Indonesia

**Keywords:** periodontitis, platelet-rich fibrin, professional mechanical plaque removal, subgingival scaling, root planing

## Abstract

**Background and Objectives:** Periodontitis is an inflammatory disease that compromises the supporting structures of the teeth, leading to irreversible tissue damage and tooth loss. While subgingival professional mechanical plaque removal (PMPR) remains the gold standard treatment, there is increasing interest in adjunctive therapies. Platelet-rich fibrin (PRF) has gained attention as a promising biomaterial to enhance periodontal healing and regeneration. This study aimed to evaluate the clinical and immunological effectiveness of PRF as an adjunct to PMPR. **Materials and Methods:** Clinical studies published between January 2019 and August 2024 were included from the ProQuest, PubMed, PMC, ScienceDirect, Scopus, and EBSCO databases. Seven studies met the inclusion criteria, focusing on adults with periodontitis treated with PRF + PMPR compared to PMPR alone. Primary outcomes included changes in clinical and immunological parameters. Risk of bias was assessed using the Cochrane ROB2 tool. Meta-analysis was conducted using both fixed-effect and random-effects models, depending on heterogeneity. **Results:** The meta-analysis demonstrated significant improvements in clinical outcomes in the PRF + PMPR group, with reductions in probing pocket depth (SMD: −1.43 mm; 95% CI: −2.05 to −0.81; *p* < 0.00001), clinical attachment level (SMD: −1.34 mm; 95% CI: −1.95 to −0.73; *p* < 0.0001), bleeding on probing (SMD: −0.75 mm; 95% CI: −1.11 to −0.39; *p* < 0.00001), gingival recession (SMD: −0.79 mm; 95% CI: −1.33 to −0.25; *p* = 0.004), and gingival index (SMD: −0.82 mm; 95% CI: −1.37 to −0.28; *p* = 0.003). Favorable trends were also observed in IL-10, TGF-β, VEGF, PDGF-BB, periostin, and type I collagen levels. **Conclusions:** PRF enhances clinical and immunological outcomes and supports periodontal tissue stability when used as an adjunct to non-surgical therapy.

## 1. Introduction

Periodontitis is a chronic oral disease linked to an imbalance in the microbial community of the mouth. This imbalance, or dysbiosis, involves a shift in the type and behavior of bacteria, allowing disease-causing species to dominate. The development and progression of periodontitis involve a continuous interaction between these harmful bacteria and an abnormal immune-inflammatory response in the host. In susceptible individuals, this leads to the destruction of the supporting structures of the teeth. In severe cases, it can result in tooth mobility, tooth loss, and a higher likelihood of systemic non-communicable diseases [1,2]. The hallmark features of periodontitis include the loss of clinical attachment and alveolar bone, which can be seen through radiographic evaluation, the presence of periodontal pockets, and gingival bleeding [3].

The World Health Organization (WHO) estimates that 19% of the global adult population suffers from severe periodontal disease [4]. According to the 2019 Global Epidemiological Study, there were 1.1 billion cases of severe periodontitis. Between 1990 and 2019, the age-standardized prevalence rate of severe periodontitis increased by 8.44% worldwide. Global population growth contributed to 67.9% of the increase in the number of severe periodontitis cases from 1990 to 2019. Population growth is identified as a primary factor in the rising global prevalence of periodontitis [5,6]. In Indonesia, the 2018 Basic Health Research (RISKESDAS) reported a periodontitis prevalence of 74.1% [7].

The progression of periodontitis is often asymptomatic. Thus, the management of periodontitis is frequently delayed, exacerbating the progression of the disease and complicating therapeutic outcomes [8]. Therefore, proactive measures to prevent the progression of periodontitis are essential within clinical practice [9]. Non-surgical periodontal therapy (NSPT) remains the gold standard of periodontitis management [10]. Professional mechanical plaque removal (PMPR) is a non-surgical procedure to control plaque and calculus accumulation. The term PMPR is a recent update by the British Society of Periodontology. It is used to define both supragingival and subgingival instrumentation, replacing scaling and root planing (SRP) as the previous term [11].

The PMPR procedure demonstrates a high success rate; however, its efficacy may be compromised in certain cases [12]. Severe periodontitis, particularly when accompanied by deep periodontal pockets, furcation involvement, or areas with complex root morphology, can result in residual subgingival biofilm within the periodontal pockets following subgingival PMPR [13]. In such instances, adjunctive therapy might be necessary [9]. Currently, various non-surgical adjunctive therapies for periodontitis are still under research.

Platelets are a specialized component of circulating blood. These cells have a lifespan of 8–10 days and contribute to multiple biological processes. Beyond their primary function in hemostasis, platelets are key mediators in wound healing, angiogenesis, and innate immune responses. Their ability to release a variety of growth factors and signaling molecules makes them a valuable tool in regenerative therapies [14,15].

In dentistry, platelets have a wide range of applications, particularly in oral and maxillofacial surgery, where they are used as grafting materials to support bone regeneration. Their use is especially valuable in procedures aimed at bone volume augmentation [15,16]. Regenerative dentistry is a rapidly evolving field that incorporates autologous biomaterials such as platelet concentrates [15,17]. Platelet concentrates are derived from whole blood through centrifugation and are widely used due to the essential role of platelets in tissue repair [18]. One of the materials that has been extensively studied and developed is platelet-rich fibrin (PRF) [19].

Platelet-rich fibrin (PRF) is a fibrin matrix enriched with growth factors that facilitate periodontal tissue regeneration [20]. The fibrin matrix in PRF acts as a scaffold, supporting cytokines—interleukin (IL)-1β, IL-6, IL-4, and tumor necrosis factor-α (TNF-α)—and growth factors such as vascular endothelial growth factor (VEGF), insulin growth factor (IGF), transforming growth factor β (TGF-β), and platelet-derived growth factor (PDGF), all of which play pivotal roles in stimulating tissue healing and periodontal regeneration [21,22]. Platelet-rich fibrin (PRF) contains a higher concentration of leukocytes compared to its predecessor, platelet-rich plasma (PRP) [23].

Unlike PRP, the preparation protocol for PRF does not require the use of anticoagulants nor bovine thrombin (nor any other gellants). This allows for the natural formation of blood clots, eliminating risks associated with the use of anticoagulants, especially bovine thrombin [21]. Following the centrifugation process, PRF spontaneously forms a dense fibrin matrix, which allows for a slower degradation rate. This facilitates the gradual release of growth factors into the tissue during the wound-healing process [21,24]. Compared to PRP, PRF is considered to have a more standardized, efficient, and cost-effective procedural protocol, while also demonstrating superior clinical outcomes [21].

PRF has shown beneficial effects when used as an adjunct in periodontal surgery. Beyond its classification as a platelet concentrate, it acts like a graft by continuously releasing growth factors for over a week. Studies on intrabony defects report that PRF enhances clinical outcomes, such as PPD reduction, CAL gain, and bone fill, especially when used similarly to guided tissue regeneration membranes [25,26,27]. PRF has also improved results in sinus floor elevation procedures and has been applied alongside implant placement to help support sinus membrane elevation. In extraction sites, PRF has been associated with reduced bone resorption and better preservation of the alveolar ridge [28].

A study by Balice et al. further supports the role of PRF in treating intrabony defects. Their findings indicate that combining PRF with autologous bone graft yields CAL gains comparable to those achieved with collagen membranes and bone grafts, but with significantly less gingival recession—an important consideration in esthetic zones such as the anterior sextants [29].

Given the beneficial effects of PRF in periodontal surgery, there is growing interest in exploring PRF as an adjunct to NSPT. Several studies have shown that incorporating PRF into non-surgical therapy protocols can mitigate the challenges and risks associated with surgical periodontal procedures, such as gingival recession, postoperative pain and swelling, and bleeding [30,31]. For example, Parwani et al. found that PRF application significantly reduced PPD, particularly in sites measuring 5–6 mm. The use of PRF in such cases may also reduce the number of sessions required to achieve pocket closure [32].

Research on the use of PRF as an adjunct to non-surgical periodontal therapy is currently limited and presents inconsistent findings. Some studies comparing subgingival PMPR with i-PRF versus PMPR alone report no significant clinical improvements [33]. The overall evidence base remains sparse and heterogeneous.

Existing studies differ widely in their assessment approaches—some prioritize clinical parameters, while others focus on immunological markers, leading to inconsistent outcome measures. In addition, PRF preparation methods vary, with PRF clots and i-PRF being the most frequently used formulations [32,34,35,36,37,38,39]. These methodological differences contribute to varied results. This review aims to evaluate the effectiveness of PRF as an adjunct to subgingival PMPR, based on clinical and immunological outcomes reported in the current literature.

## 2. Materials and Methods

### 2.1. Methodology

The selection of the study was performed according to the Preferred Reporting Items for Systematic Reviews and Meta-Analyses (PRISMA) guidelines. This research is a property registered with the International Prospective Register of Systematic Reviews (PROSPERO) under the registration number CRD42024592058. 

### 2.2. Search Strategies

We performed a systematic review of clinical studies that assessed the beneficial effect of PRF as an adjunctive non-surgical therapy following subgingival PMPR in periodontitis. Publications written in English from January 2019 to August 2024 were included. The selected studies were identified by searching six electronic databases, namely, ProQuest (www.proquest.com), PubMed (pubmed.ncbi.nlm.nih.gov, accessed on 31 August 2024), PMC (www.ncbi.nlm.nih.gov/pmc/, accessed on 31 August 2024), ScienceDirect (sciencedirect.com/, accessed on 31 August 2024), Scopus (www.scopus.com, accessed on 31 August 2024), and EBSCO (web.p.ebscohost.com, accessed on 31 August 2024). The search was last conducted on 31 August 2024. The search strategy employed Boolean operators to construct the following search string: (“Platelet-Rich Fibrin” OR “PRF”) AND (“Periodontitis” OR “Periodontal Disease”) AND (“Professional Mechanical Plaque Removal” OR “PMPR” OR “Subgingival Instrumentation” OR “Scaling and Root Planing” OR “SRP”). Database search queries are presented in Supplementary Materials Table S1.

This research was conducted based on the question, “What is the beneficial effect of PRF as an adjunctive non-surgical therapy for periodontitis?” The research question is outlined using the PICO-S framework, which consists of population, intervention, comparison, outcome, and study design as described in Table 1.

### 2.3. Inclusion Criteria

Clinical studies had to meet the following criteria: (1) adult patients ≥18 years with a diagnosis of periodontitis, (2) clinical studies evaluating the beneficial effect of PRF as an adjunctive non-surgical therapy following subgingival PMPR in periodontitis, compared to subgingival PMPR alone, (3) studies reporting clinical parameters (PPD, CAL, BoP, GR, GI, PlI) and/or immunological markers (IL, TNF-α, VEGF, TGF-β, PDGF, periostin, collagen type I), (4) studies published in English within the last five years (January 2019–August 2024), (5) clinical studies, including RCTs, pilot studies, comparative studies, and case reports, (6) full-text articles available.

### 2.4. Exclusion Criteria

Exclusion criteria in this systematic review and meta-analysis include: (1) systematic review and meta-analyses, animal studies, and in vitro studies, (2) studies involving additional therapy besides PRF, (3) studies with subjects who have systemic diseases, and (4) studies with subjects who smoke or consume alcohol.

### 2.5. Data Extraction

Articles were collected and managed with Zotero, then imported to the Rayyan Intelligent Systematic Review. Data extraction involved labeling and clustering similar studies that met the predefined inclusion criteria. Initial information (titles and abstracts) extracted from the articles was verified by a single reviewer (M.T.). The retrieved studies were then further validated by two reviewers (F.M.T., S.L.C.M). Final inclusion of studies was confirmed by three independent reviewers working separately to ensure objectivity and consistency (M.T., F.M.T., S.L.C.M.). Data were extracted from all eligible studies using a standardized key for characteristics: periodontitis; platelet-rich fibrin; professional mechanical plaque removal; subgingival instrumentation; follow-up intervals; variables measured (clinical/immunological parameters). In instances of missing data, we attempted to contact the corresponding authors to obtain the necessary information.

The data from the selected articles was compiled into a Microsoft Excel spreadsheet. Data extracted from included studies were (1) first author and year of publication, (2) country of conduct, (3) study design, (4) total samples and sites, (5) age of samples, (6) definition and criteria of periodontitis, (7) intervention, (8) outcome variables, including clinical and immunological parameters, and (9) follow-up periods of each parameters. Protocols of PMPR procedures and PRF application were also retrieved from each study.

### 2.6. Risk of Bias Assessment

The risk of bias in this systematic review was assessed using the Cochrane Risk of Bias 2 (ROB 2) tool, specifically designed to evaluate bias in randomized trials. Cochrane ROB 2 evaluates five domains: bias arising from the randomization process (D1), bias due to deviations from the intended interventions (D2), bias due to missing outcome data (D3), bias in the measurement of the outcome (D4), and bias in the selection of the reported results (D5). The assessment was carried out by three independent reviewers (M.T., F.M.T., N.A.H.). Each reviewer conducted the assessment separately and discussed their findings to reach a consensus on the risk of bias for each study. The RoB 2 assessment then placed the studies into three categories: low risk, some concerns, or high risk. Funnel plots were used to assess the risk of publication bias and are presented in the Supplementary Materials Figures (Figures S1–S6).

### 2.7. Statistical Analysis

Post-intervention continuous outcomes across study groups were combined using the standard mean difference, accompanied by 95% confidence intervals (CI). For meta-analysis, ≥2 studies per outcome were required for pooling. Results are presented in the form of a forest plot generated by RevMan 5.4.1 software. The forest plot was utilized to illustrate the strength of the association between the test group (PRF + PMPR) and the control group (PMPR only). A Z-test is employed to determine whether the null hypothesis can be rejected, assuming a normally distributed random-effects model for effect sizes. A *p*-value < 0.05 indicates a rejected null hypothesis, showing the significance of the intervention. Heterogeneity among studies is assessed using the I^2^ statistic. As a general guideline, an I^2^ value below 50% indicates acceptable heterogeneity, while values exceeding 50% suggest potential inconsistency among studies. Random-effects models were applied when heterogeneity exceeded 50%. Sensitivity analyses were performed to explore potential sources of heterogeneity. The GRADE summary of findings is presented in Supplementary Materials Table S2 as a statement on the certainty of evidence. When data did not meet the criteria for meta-analysis, a qualitative (narrative) synthesis was conducted and further discussed in the results and discussion sections.

## 3. Results

### 3.1. Characteristics of the Included Studies

**Figure 1:**
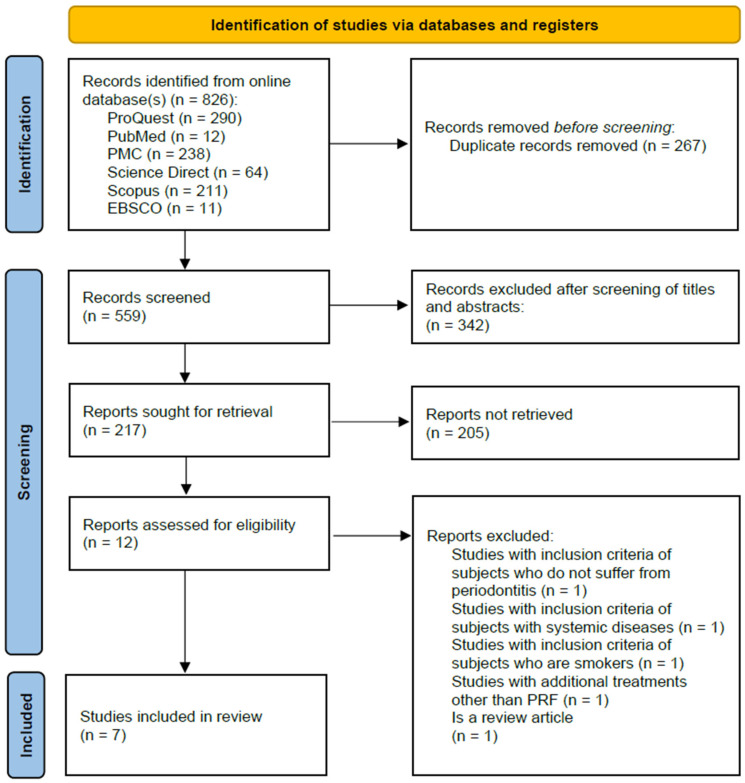
PRISMA 2020 flow diagram.

The study selection process is illustrated in the PRISMA flow diagram (Figure 1). A total of 826 studies were retrieved from six electronic databases: ProQuest (*n* = 290), PubMed (*n* = 12), PMC (*n* = 238), ScienceDirect (*n* = 64), Scopus (*n* = 211), and EBSCO Medline (*n* = 11). The reference management tool Rayyan was utilized to facilitate study synthesis and screening. After the removal of 267 duplicate records, 559 unique studies remained for initial screening. Based on title and abstract screening, 12 studies were identified as potentially eligible. Following full-text assessment, five studies were excluded for not meeting the predefined inclusion criteria. Consequently, seven studies were included in the systematic review [32,34,35,36,37,38,39], of which six studies [34,35,36,37,38,39] were eligible for inclusion in the quantitative meta-analysis.

Seven studies were randomized, including six clinical trials [32,34,35,37,38,39] and one pilot study [36] with the use of split-mouth trials (Table 2). Sample sizes ranging from 12 to 24 participants, both male and female, are included. Adult populations were targeted for these studies, with participants ranging in age from 20 to 64 years. The subjects were patients with periodontitis, defined using varying criteria across studies, although a common standard was a probing pocket depth (PPD) of ≥5 mm. These seven studies analyze the outcomes between PRF as an adjunct to PMPR and PMPR alone in different outcome variables, such as clinical and immunological parameters. Each of these studies also shows different methods in PRF preparation. Follow-up intervals varied between 1–6 months for clinical parameters and three days to three months for immunological parameters. Immunological parameters were typically assessed on the 7th and 14th days, as PRF can release growth factors at high concentrations throughout that time frame [40].

At the outset, most studies provided participants with oral health education and plaque control training. Some participants also underwent supragingival PMPR. A randomized split-mouth design was used, in which each participant received both the control treatment (PMPR only) and the intervention (PMPR + PRF). Two studies reported the use of local anesthesia before PMPR. Instruments used included Gracey curettes and/or ultrasonic scalers. Platelet-rich fibrin was prepared using a centrifugation system, with variations in speed and duration (Table 3).

### 3.2. Risk of Bias

A study conducted by Parwani et al. exhibited some concerns regarding bias. These concerns were attributed to limited information related to the operator’s blinding to the interventions administered to the subjects. Aside from this issue, the cumulative assessment indicates low risk of bias. Deviations from the intended interventions were minimal across all studies, with no notable concerns regarding missing outcome data. Outcome measurements were consistently reliable, and there was no indication of selective outcome reporting. Notably, 85% of studies were evaluated as having a low risk of bias.

**Figure 2:**
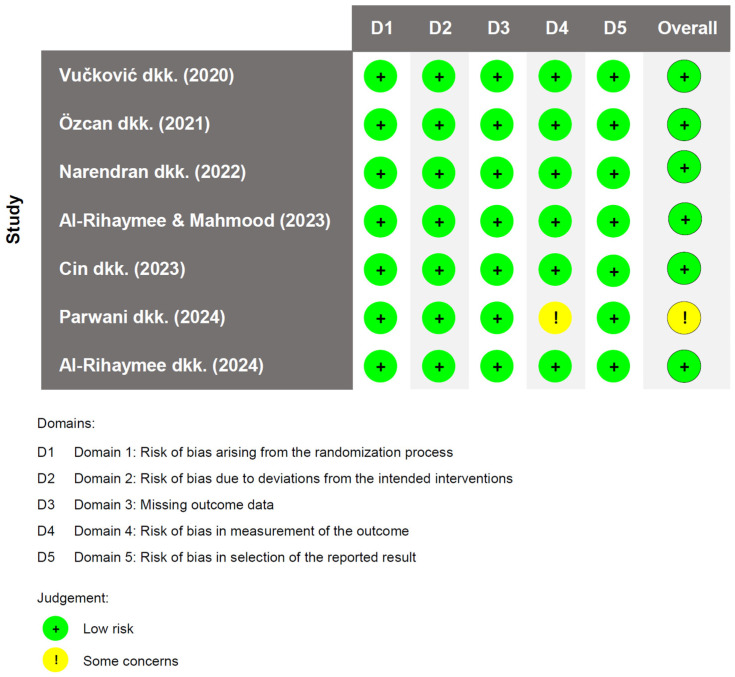
Cochrane RoB 2 assessment [32,34,35,36,37,38,39].

Funnel plots were generated to visually assess potential publication bias for the primary outcomes (Supplementary Materials Figures S1–S6). The funnel plots for BoP, GI, and PlI appeared relatively symmetrical, indicating a low risk of publication bias. In contrast, slight asymmetry was observed in the plots for PPD, CAL, and GR, with most studies clustering on the left side of the pooled effect line. This distribution suggests a potential favorable effect of the intervention compared to the control. However, the limited number of studies included in the meta-analysis reduces the reliability of these assessments. The risk of bias assessment is presented in Figure 2 as follows.

### 3.3. Assessment of Certainty

Certainty of the evidence was assessed using the GRADE approach. Summary ratings of evidence quality are provided in the Supplementary Materials Table S2 and reflect considerations such as limitations, inconsistency, and imprecision. Overall, the evidence was rated as ranging from low to moderate certainty, primarily due to limited study numbers. Notable inconsistencies were found for PPD and CAL due to high heterogeneity.

### 3.4. Qualitative Analysis

We evaluate the beneficial effect of PRF as an adjunctive NSPT following subgingival PMPR. The components of PRF are believed to accelerate the healing process. This healing is assessed through clinical and immunological parameters, which serve as indicators of periodontitis. This systematic review utilized various studies that analyzed clinical and immunological parameters as their variables. The analysis included seven clinical studies in total (six randomized clinical studies and one randomized pilot study). Data summary of clinical and immunological parameters from the included studies is presented in Supplementary Materials Tables S3 and S4.

### 3.5. Quantitative Analysis

Clinical parameters were quantitatively analyzed and illustrated using forest plots to compare the test and control groups before and three months after PRF therapy. While some studies reported additional follow-up periods (1, 1.5, 2, and 6 months), the data were insufficient for meta-analysis at those time points. Notably, the three months follow-up is widely regarded as the optimal interval for evaluating periodontal therapy outcomes [33]. In terms of immunological parameters, there was insufficient clinical research for determining the success of periodontal therapy. Therefore, meta-analysis was performed on six studies that present changes in clinical parameters (PPD, CAL, BoP, GR, GI, and PlI).

#### 3.5.1. Probing Pocket Depth (PPD)

**Figure 3:**
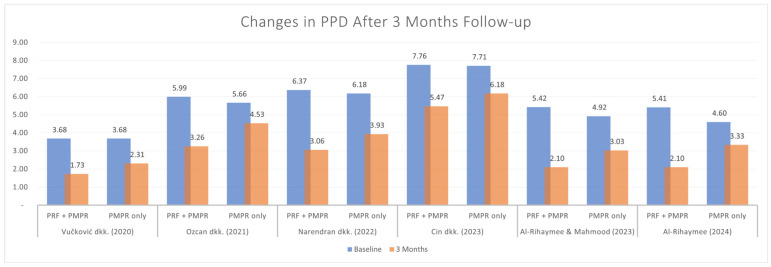
Changes in PPD after three months of therapy [34,35,36,37,38,39].

**Figure 4:**
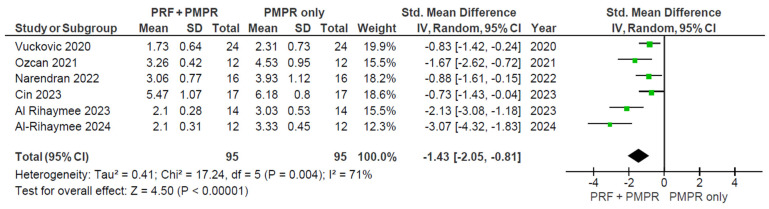
Meta−analysis of PPD before and after three months of therapy. The effect size of each study [34,35,36,37,38,39] is represented by the green squares. Pooled size effect is represented by the black diamond. Horizontal lines indicate 95% confidence intervals. Vertical line indicates line of no effect.

All included studies provided sufficient data for the meta-analysis of probing pocket depth (PPD). Both test and control groups showed reductions in PPD, with a greater reduction observed in the test group, as illustrated in Figure 3 and Figure 4. The pooled mean difference was −1.43 mm (95% CI: [−2.05; −0.81]; *p* < 0.00001), favoring the PRF group. Despite the statistically significant result, high heterogeneity was present, prompting the use of a random-effects model. This limitation is addressed further in the discussion.

**Figure 5:**
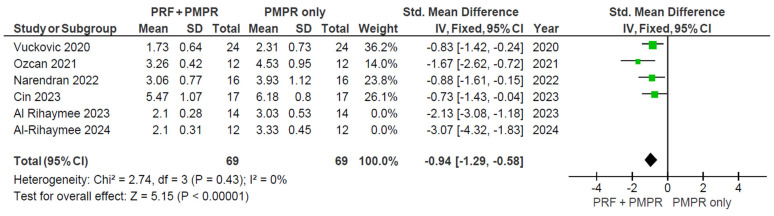
Sensitivity analysis of PPD. The effect size of each study [34,35,36,37,38,39] is represented by the green squares. Pooled size effect is represented by the black diamond. Horizontal lines indicate 95% confidence intervals. Vertical line indicates line of no effect.

Sensitivity analysis was used to assess the robustness and reliability of the overall findings. In this study, a sensitivity analysis (Figure 5) was done by removing studies with larger effect sizes (Al-Rihaymee and Mahmood, 2023 [38], Al-Rihaymee et al., 2024 [37]), possibly from clinical effect differences.

#### 3.5.2. Clinical Attachment Loss (CAL)

**Figure 6:**
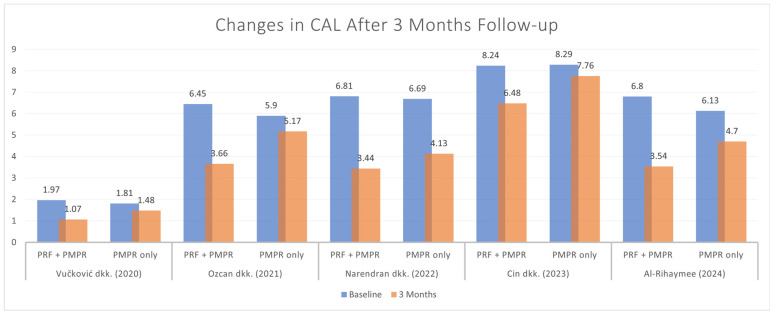
Changes in CAL before and after three months of therapy [34,35,36,37,39].

**Figure 7:**
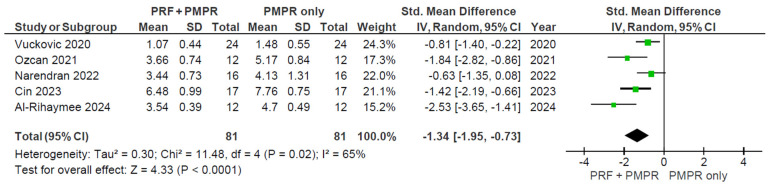
Meta−analysis of CAL after three months of therapy. The effect size of each study [34,35,36,37,39] is represented by the green squares. Pooled size effect is represented by the black diamond. Horizontal lines indicate 95% confidence intervals. Vertical line indicates line of no effect.

Five of six studies provided sufficient data for the meta-analysis of clinical attachment level (CAL). Both groups showed significant CAL reductions, with the test group demonstrating a greater improvement (Figure 6). The pooled analysis (Figure 7) revealed a statistically significant difference favoring the test group, with a mean difference of −1.34 mm (95% CI: [−1.95; −0.73], *p* < 0.0001). Given the moderate heterogeneity (I^2^ = 65%), a random-effects model was used. These findings suggest that PRF, when used alongside subgingival PMPR, significantly improves CAL in non-surgical periodontal therapy.

**Figure 8:**
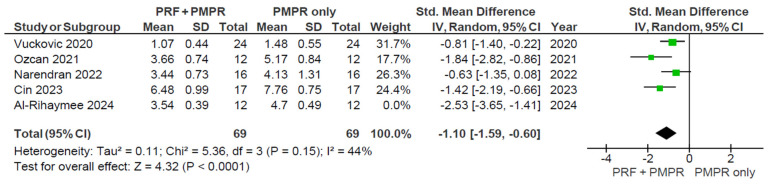
Sensitivity analysis of CAL. The effect size of each study [34,35,36,37,39] is represented by the green squares. Pooled size effect is represented by the black diamond. Horizontal lines indicate 95% confidence intervals. Vertical line indicates line of no effect.

A sensitivity analysis for CAL was performed by removing the outlier study (Al-Rihaymee et al., 2024 [37]) in Figure 8, which reported an unusually large effect size and a small standard deviation. This outlier may reflect true clinical differences or methodological variations.

#### 3.5.3. Bleeding on Probing (BoP)

**Figure 9:**
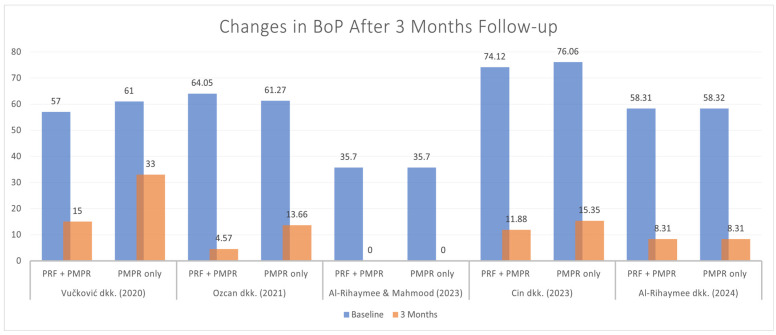
Changes in BoP after three months of therapy [34,35,37,38,39].

**Figure 10:**
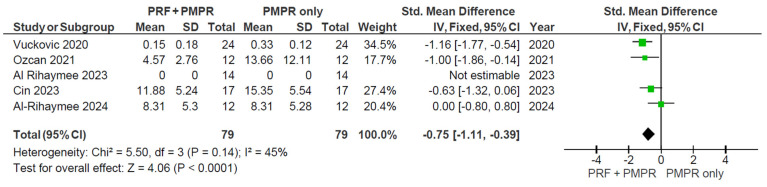
Meta−analysis of BoP before and after three months of therapy. The effect size of each study [34,35,37,38,39] is represented by the green squares. Pooled size effect is represented by the black diamond. Horizontal lines indicate 95% confidence intervals. Vertical line indicates line of no effect.

Five of six studies provided data for the meta-analysis of bleeding on probing (BoP). Both test and control groups showed significant reductions, with a greater reduction observed in the test group (Figure 9). The pooled mean difference was −0.75 mm (95% CI: [−1.11, −0.39]; *p* < 0.00001), favoring the use of PRF (Figure 10). These findings suggest that PRF as an adjunct to subgingival PMPR significantly improves BoP reduction in non-surgical periodontal therapy.

#### 3.5.4. Gingival Recession (GR)

**Figure 11:**
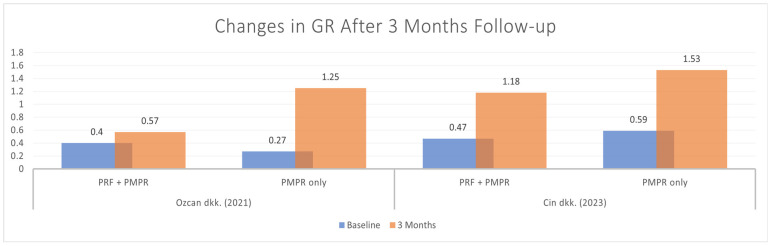
Changes in GR after three months of therapy [35,39].

**Figure 12:**
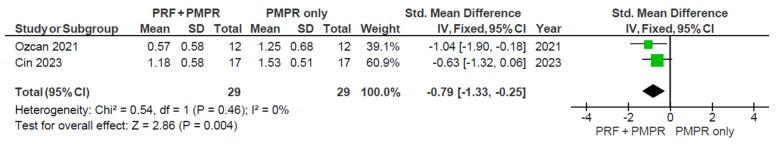
Meta−analysis of GR before and after three months of therapy. The effect size of each study [35,39] is represented by the green squares. Pooled size effect is represented by the black diamond. Horizontal lines indicate 95% confidence intervals. Vertical line indicates line of no effect.

Only two of six studies provided sufficient data for the meta-analysis of gingival recession (GR). Both groups showed an improvement on GR, with a greater result in the test group, as illustrated in Figure 11. The pooled mean difference was −0.79 mm (95% CI: [−1.33; −0.25]; *p* = 0.004), indicating a statistically significant improvement in favor of PRF (Figure 12). These findings suggest that PRF as an adjunct to subgingival PMPR significantly improves GR in non-surgical periodontal therapy.

#### 3.5.5. Gingival Index (GI)

**Figure 13:**
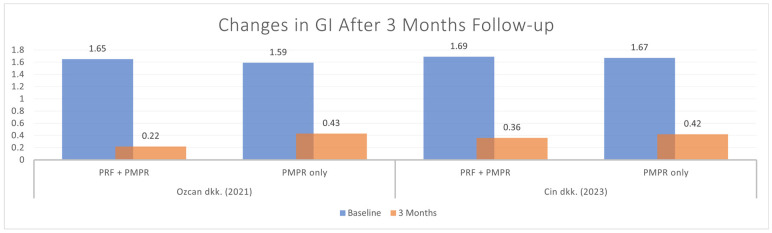
Changes in GI after three months of therapy [35,39].

**Figure 14:**
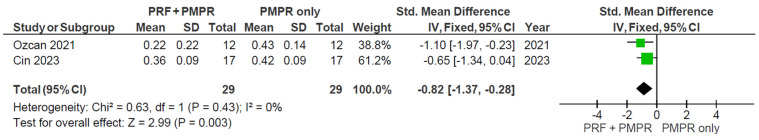
Meta−analysis of GI before and after three months of therapy. The effect size of each study [35,39] is represented by the green squares. Pooled size effect is represented by the black diamond. Horizontal lines indicate 95% confidence intervals. Vertical line indicates line of no effect.

Out of six studies, two provided sufficient data for GI meta-analysis. Both showed significant GI reduction in the test and control groups (Figure 13). As shown in Figure 14, the pooled analysis revealed a significant difference favoring the test group, with a mean difference of −0.82 mm (95% CI: [−1.37, −0.28], *p* = 0.003). These findings support the effectiveness of PRF as an adjunct to subgingival PMPR in improving gingival health.

#### 3.5.6. Plaque Index (PlI)

**Figure 15:**
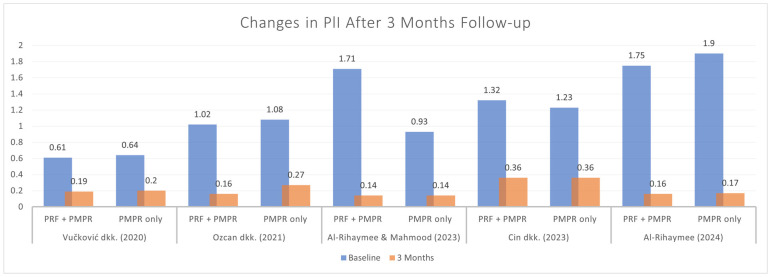
Changes in PlI after three months of therapy [34,35,37,38,39].

**Figure 16:**
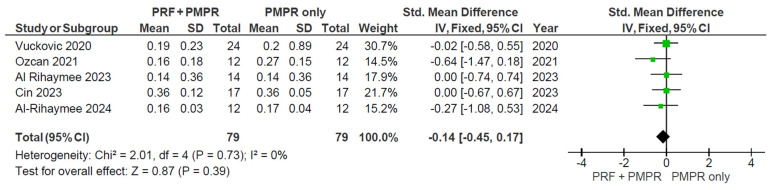
Meta−analysis of PlI before and after three months of therapy. The effect size of each study [34,35,37,38,39] is represented by the green squares. Pooled size effect is represented by the black diamond. Horizontal lines indicate 95% confidence intervals. Vertical line indicates line of no effect. The black diamond crosses the line of no effect, meaning that the role of PRF remains insignificant in reducing PlI.

Five of six studies provided data for the PlI meta-analysis. Both groups showed PlI reduction (Figure 15), but the pooled analysis (Figure 16) found no significant difference between them (SMD: −0.14 mm; 95% CI: [−0.45, 0.17]; *p* = 0.39). Thus, PRF as an adjunct to NSPT after subgingival PMPR showed no significant impact on PlI.

## 4. Discussion

### 4.1. Beneficial Effect on Clinical Parameters

The use of PRF as an adjunctive non-surgical therapy after subgingival PMPR in periodontitis demonstrated improvements in clinical parameters, particularly in PPD reduction. All seven studies consistently showed lower final PPD values in the PRF + PMPR group compared to PMPR alone [32,34,35,36,37,38,39]. The meta-analysis supports these findings, though high heterogeneity—likely due to varying baseline PPD scores—was observed [34,35,36,37,38,39]. PRF’s effectiveness may be linked to its immunological and antibacterial properties, including cytokine-induced angiogenesis and inflammation modulation. A systematic review by Pullishery et al. also found i-PRF more effective in reducing PPD than xenografts and hydroxyapatite grafts [41].

The use of PRF as an additional non-surgical therapy after subgingival PMPR also significantly reduced CAL values. Six out of seven studies showed significant CAL reduction when compared between test and control groups [32,34,35,36,37,39]. The high heterogeneity observed in the meta-analysis is largely attributable to differences in baseline clinical attachment level (CAL) scores among the included studies. This inconsistency stems from variations in the definitions and diagnostic criteria for periodontitis. In addition, the studies varied in terms of population characteristics (gender and age distribution) and clinical settings, which further contributed to the observed heterogeneity.

While PPD and CAL levels showed significant differences between test and control groups, BoP percentages did not consistently follow this trend. Only one of five studies reported a significant BoP reduction [34]. A meta-analysis revealed significant differences, likely due to BoP increases at six months after earlier reductions. For instance, Vučković et al. observed a drop from 57% to 15% [34] and Özcan et al. reported a decrease from 64% to 4.57% at three months, followed by an increase at six months [35]. Similarly, Cin et al. noted a decline at one month, but BoP rose again by the third and sixth months [39].

The increase in BoP at the six-month post-treatment follow-up may be related to an increase in the composition of periodontopathogenic bacteria. Soeroso et al. noted the significant growth of *Porphyromonas gingivalis*, *Tannerella forsythia*, and *Treponema denticola* at the six-month mark after subgingival PMPR. While subgingival PMPR is effective in temporarily reducing bacterial count, it is not sufficient to control the regrowth of these periodontopathogenic bacteria without additional periodontal treatment over six months. Periodontitis patients remain at higher risk of recurrence compared to gingivitis patients or healthy individuals [42]. Therefore, managing periodontitis requires continuous risk assessment as part of optimal patient care management [43].

Gingival recession (GR) often follows periodontal surgery due to root exposure and reduced inflammation [44]. Özcan et al. suggested that non-surgical approaches may better preserve gingival volume. Both Özcan et al. and Cin et al. reported significant GR increases in test and control groups over six months [35,39], while Parwani et al. found minimal GR in both groups at 1.5 months post-treatment [32]. Differences in findings may stem from measurement methods: Özcan et al. and Cin et al. used gingival margin height (higher values indicating more recession), whereas Parwani et al. measured from the cementoenamel junction to the gingival margin (lower values indicating improvement). [35,39]. A meta-analysis confirmed significant differences in GR outcomes, supporting these findings.

All studies assessing GI and PlI used the Silness and Löe scoring system. Özcan et al., Cin et al., and Parwani et al. reported consistent post-treatment decreases in GI following PRF and subgingival PMPR [32,35,39]. Meta-analysis showed significant GI improvement at three months, but not at six months, likely due to suboptimal maintenance. For PlI, the meta-analysis found no statistically significant differences. Vučković et al. observed a marked PlI reduction in both groups after three months, but no difference between them [34]. Özcan et al. noted a decrease at three months, followed by an increase at six [35]. Variability in PlI outcomes appears linked to patient adherence. Follow-up schedules and oral hygiene reinforcement varied across studies, affecting consistency. Due to these compliance factors, it is unclear if PRF directly impacts PlI.

### 4.2. Beneficial Effect on Periodontal Stability

The 2017 World Workshop defined periodontal treatment success based on PPD and BoP: (1) stable periodontitis with BoP < 10% and PPD ≤ 4 mm, (2) stable with gingival inflammation (BoP > 10%), and (3) unstable with PPD ≥ 4 mm and BoP [9,36]. Five of seven studies assessed both parameters over a three-month follow-up to evaluate PRF’s effect on stability [34,35,37,38,39]. In the study by Vučković et al., the test group experienced a decrease in PPD to 1.73 ± 0.64 mm with a BoP of 15% after three months, which categorized them as stable periodontitis patients with gingival inflammation. Vučković et al. reported a test group PPD of 1.73 mm and BoP of 15%, indicating stability with inflammation [34]. Özcan et al. found PPD of 3.26 mm and BoP of 4.57%, while Al-Rihaymee and Mahmood observed PPD of 2.10 mm and 0% BoP—both classified as stable [35,38]. Cin et al. reported PPD of 5.47 mm and BoP of 11.88%, indicating instability [39]. Another Al-Rihaymee study showed PPD of 2.10 mm and BoP of 8.31%, also stable [37]. Overall, three out of five studies demonstrated periodontal stability with PRF, suggesting its benefit in maintaining tissue health.

### 4.3. Beneficial Effect on Immunological Parameters

Four of seven studies examined immunological markers, including those by Özcan et al., Al-Rihaymee and Mahmood, Cin et al., and Al-Rihaymee et al. Cin et al. reported a significant increase in IL-10 levels in the test group’s GCF after subgingival PMPR, likely due to i-PRF’s sustained anti-inflammatory effects [39]. Emingil et al. found that subgingival PMPR raised IL-4, IL-10, IL-13, and IL-17 levels, improving periodontal status [45]. Passoja et al. reported higher IL-10 levels in healthy individuals and a negative correlation between IL-10 and BoP, PPD, and CAL [46].

Cin et al. reported a significant reduction in TNF-α levels in the test group compared to the control group at each follow-up, likely due to i-PRF’s anti-inflammatory and growth factor-releasing effects [39]. Zhang et al. found that i-PRF was able to suppress dendritic cell activation, which plays a role in the pathophysiology of periodontitis, as well as the polarization of M1 macrophages. Additionally, i-PRF inhibits inflammatory factors such as toll-like receptor 4 (TLR4) and phosphatase 6 protein, which are key factors in the NF-κB signaling pathway associated with inflammation [47].

VEGF is a glycoprotein that regulates angiogenesis, endothelial cell activity, and vascular permeability, playing a key role in blood vessel formation [21,48]. Cin et al. found that VEGF levels in GCF were lower on day 7 than on day 14 post-treatment in both groups. However, by day 14, the test group showed significantly higher VEGF levels, indicating the initiation of wound healing. The initial decrease in VEGF levels on day 7 may reflect reduced inflammation following subgingival PMPR, with i-PRF’s fibrin matrix promoting VEGF release [39].

TGF-β plays a key role in wound healing, tissue regeneration, and inflammation regulation, stimulating fibroblast and osteoblast proliferation while inhibiting epithelial cell growth [21,22,35]. Özcan et al. found significantly higher TGF-β levels in the test group compared to the control on days 3, 7, and 14. The decrease in TGF-β levels in both groups indicated inflammation resolution, while the increase in the test group suggests PRF’s regenerative potential during early wound healing, particularly up to day 14 [35].

A study of PDGF-BB was undertaken by Al-Rihaymee et al. The study found a significant increase in PDGF-BB levels in GCF during the first- and third-month follow-ups for both test and control groups. This increase is likely due to PRF’s effect on alveolar bone regeneration and its role in enhancing blood supply to soft tissue and underlying bone. PDGF-BB promotes angiogenesis, stem cell differentiation, and tissue repair by stimulating cell migration and mitogenesis at injury sites [29,42]. Platelet-derived growth factor-BB (PDGF-BB) subsequently promotes angiogenesis, chemotaxis, and mitogenesis at the target sites [22,37]. While PDGF-BB release is highest in the first ten days, it continues to rise consistently through the third month, as reported by Al-Rihaymee et al. [37].

Al-Rihaymee and Mahmood found significantly higher periostin levels in the test group compared to the control group at one and three months. Periostin increased from 26.35 ± 6.53 ng/μL at baseline to 48.83 ± 9.3 ng/μL at 1 month, and 98.90 ± 24.94 ng/μL at three months. The low baseline periostin levels reflected inflammation-related tissue damage. Following inflammation resolution, periostin is released during the formation of the periodontal ligament and periosteum. TGF-β and BMP-2 induce periostin expression in gingival fibroblasts, while TNF-α inhibits it [30,43]. Periostin strengthens collagen cross-linking, enhancing tissue mechanics, which explains its increase after periodontal therapy.

Özcan et al. found higher levels of type I collagen at the test site compared to the control at all measurement points, particularly on days 3 and 7. This increase was closely linked to elevated TGF-β levels, as TGF-β in the PRF matrix regulates type I collagen in periodontal tissues [35]. Hou et al. showed that TGF-β gene transfer into stem cells accelerates periodontal ligament regeneration by upregulating type I and III collagen [49]. The decrease in type I collagen on day 14 likely reflects optimal regulation by day 7 [35].

This study highlights the complex interplay of immunological parameters in periodontal healing. TGF-β supports collagen type I and periostin expression, essential for connective tissue formation and wound healing. Its increase is accompanied by higher periostin levels, reflecting tissue repair and strengthened periodontal structures [35]. TGF-β also works with IL-10, an anti-inflammatory cytokine that aids inflammation resolution and healing [50]. In contrast, the decrease in TNF-α, a pro-inflammatory cytokine, indicates reduced inflammation and improved periodontal condition [46,51]. The balance between TGF-β, IL-10, and TNF-α plays a key role in healing, suggesting that PRF therapy supports tissue repair and inflammation control in periodontitis patients [39].

### 4.4. Limitations, Implications, and Recommendations for Future Research

This systematic review and meta-analysis have several limitations. Studies by Özcan et al., Al-Rihaymee and Mahmood, Narendran et al., and Al-Rihaymee et al. faced challenges placing PRF clots into deep, narrow periodontal pockets, making it difficult to assess how long PRF remains in the pockets and its effectiveness [35,36,37,38]. This limitation can be addressed by dividing PRF into small fragments to enhance its stability within the periodontal pocket [35,36,37,38]. Patients were also instructed not to brush their teeth on the first day after PRF application [35,38]. Membrane stability can also be enhanced by suturing the periodontal pocket, as demonstrated by Parwani et al. [32]. Two studies, Vučković et al. and Cin et al., used i-PRF [34,39], which has a more evenly distributed composition and better growth factor release control, making it a promising alternative for non-healing defects [52]. However, comparing PRF clots to i-PRF was challenging, as both showed similar clinical and immunological changes, and the studies included participants with varying periodontitis criteria and initial conditions.

Parwani et al. and Cin et al. highlighted the importance of histological studies to clarify the healing mechanisms associated with PRF therapy, specifically, whether the observed clinical improvements reflect true periodontal regeneration or merely the formation of a long junctional epithelium [32,39]. While PRF is intended to promote regeneration of cementum and periodontal ligament, epithelial migration along the root surface generally occurs more rapidly than the regeneration of connective tissues and cementum. As such, the development of a long junctional epithelium extending to the periodontal ligament level is considered undesirable [53]. However, none of the included studies provided histological data. Consequently, it remains uncertain whether the clinical gains observed represent genuine tissue regeneration or reparative healing.

The use of PRF as an adjunct to NSPT has been associated with improvements in both clinical and immunological parameters. While statistically significant gains were observed in most clinical outcomes, no notable difference was found in the PlI, indicating that plaque control is more likely influenced by patient compliance with oral hygiene and follow-up care rather than by adjunctive PRF application.

The immunological profile of periodontal tissues—evaluated via saliva and GCF biomarkers—remains highly variable across studies. This variability complicates efforts to standardize predictive markers of disease progression and limits the ability to make direct quantitative comparisons of PRF’s efficacy across different trials.

A major limitation lies in the heterogeneity of PRF protocols. Studies differ in the type of PRF used (PRF and i-PRF), follow-up durations (ranging from 1 to 6 months for clinical parameters and three days to three months for immunological parameters), outcome measurement techniques (e.g., gingival recession measured from the cementoenamel junction versus the gingival margin), and diagnostic criteria for periodontitis. Additionally, most studies were conducted in Asia and the Middle East, limiting the global applicability of findings. Small sample sizes in several studies further reduce statistical power and generalizability. Only three of the seven included studies had follow-up periods extending to six months, leaving a gap in long-term data, particularly regarding tissue stability. Although PRF shows promising benefits as an adjunctive non-surgical therapy, current evidence is still in its early stages. Larger, well-designed clinical trials with standardized protocols and extended follow-up periods are essential to establish the long-term efficacy and consistency of PRF in periodontal therapy.

## 5. Conclusions

In conclusion, studies on PRF as an adjunctive non-surgical therapy after subgingival PMPR suggest that the addition of PRF is beneficial to improve clinical and immunological parameters, as well as maintain periodontal tissue stability post-treatment. Platelet-rich fibrin (PRF) releases anti-inflammatory cytokines and growth factor levels in defective periodontal tissue, leading to more optimal results.

## Figures and Tables

**Table 1:** PICO-S Framework.

Population (P)	Adult patients (>18 years) with periodontitis
Intervention (I)	Subgingival PMPR + PRF as an adjunctive NSPT for periodontitis
Comparison (C)	Subgingival PMPR alone as a control measure
Outcome (O)	Outcome measures were clinical parameters (PPD/CAL/BoP/GR/GI/PlI) and/or immunological parameters (IL/TNF-α/VEGF/TGF-β/Periostin/Collagen type I)
Study design (S)	Clinical studies (RCT, pilot study, comparative study, case report)

**Table 2:** Overview of included studies (2020–2024).

Author (Year)	Samples Characteristics		Definition/ Diagnosis of Periodontitis	Intervention	Parameters	Follow-Up Periods
	Size	Age (Mean ± SD)				
Vučković et al., 2020 [34]	24; 10 males, 14 females	22–64 (37.29 ± 10.23)	Chronic periodontitis (minimum two teeth in each quadrant with PPD ≥ 5 mm; BoP ≥ 40%; no furcation involvement)	Subgingival PMPR + i-PRF	Clinical: PPD, CAL, BoP, GML, PlI	Baseline 3 months
Özcan et al., 2021 [35]	12; 6 males, 6 females	30–57 (43.33 ± 8.34)	Stage III grade B periodontitis (PPD ≥ 6 mm, CAL ≥ 5 mm, radiographic bone loss extending to the middle or apical third of the root, and tooth loss due to periodontitis ≤ 4 in different quadrants)	Subgingival PMPR + PRF	Clinical: PPD, CAL, BoP, GR, GI, PlI Immunological: TGF-β Col-1	Clinical: Baseline 3 months 6 months Immunological: Baseline 3 days 7 days 14 days
Narendran et al., 2022 [36]	16	35–45 (40.56 ± 3.39)	Moderate periodontitis; stage III (PPD ≥ 5 mm and ≤ 7 mm)	Subgingival PMPR + PRF	Clinical: PPD, CAL	Baseline 60 days 90 days
Al-Rihaymee & Mahmood, 2023 [38]	14; 12 males, 2 females	NR *	Periodontitis (two contralateral pockets with a depth of 4–6 mm)	Subgingival PMPR + PRF	Clinical: PPD, RAL, BoP, PlI Immunological: Periostin	Clinical: Baseline 1 month 3 months Immunological: Baseline 1 month 3 months
Cin et al., 2023 [39]	17; 7 males, 10 females	37.41 ± 5.84	Stage III grade B periodontitis (PPD ≥ 6 mm, CAL ≥ 5 mm, radiographic bone loss extending to the middle or apical third of the root, and tooth loss due to periodontitis ≤ 4 in different quadrants)	Subgingival PMPR + i-PRF	Clinical: PPD, CAL, BoP, GR, GI, PlI Immunological: VEGF TNF-α IL-10	Clinical: Baseline 1 month 3 months 6 months Immunological: Baseline 7 days 14 days
Parwani et al., 2024 [32]	13; 6 males, 7 females	30–60 (29.5)	Stage III grade A periodontitis with 5–6 mm pocket	Subgingival PMPR + PRF	Clinical: PPD, CAL, GR, GI, PlI	Baseline 6 weeks
Al-Rihaymee et al., 2024 [37]	12; 9 males, 3 females	20–40 (29.83 ± 5.7)	Unstable periodontitis with PPD 4–5 mm on both sides	Subgingival PMPR + PRF	Clinical: PPD, CAL, BoP, PlI Immunological: PDGF-BB	Clinical: Baseline 1 month 3 months Immunological: Baseline 1 month 3 months

* NR = not reported.

**Table 3:** Procedure for PMPR and PRF preparation.

Author (Year)	Pre-Intervention	PMPR Procedure			PRF Procedure				
		Anesthesia	Method	Instrument	Type	Tools	Duration	Speed	RCF
Vučković et al., 2020 [34]	Self-performed plaque control (brushing, interdental cleaning	Yes	FMS	NR	i-PRF	Duo Centrifuge (Process for PRF) Nice, France	3 min	700 rpm	60 g
Özcan et al., 2021 [35]	Manual scaling (Gracey curette) and ultrasonic, oral health education	NR	FMS	Standard curettes	PRF clot	Nuve, CN 180, Bench-Top Centrifuge Ankara, Turkey	10 min	3000 rpm	400 g
Narendran et al., 2022 [36]	NR	NR	FMS	NR	PRF clot	Remi R-8c BL Centrifugation System Mumbai, India	12 min	2700 rpm	NR
Al-Rihaymee & Mahmood, 2023 [38]	Oral hygiene education, ultrasonic scaling	NR	FMS	NR	PRF clot	Intraspin Centrifuge Boca Raton, FL, USA	10 min	3000 rpm	805 g
Cin et al., 2023 [39]	Supragingival scaling, oral hygiene education	Yes	FMS	Gracey curettes	i-PRF	Duo Centrifuge (Process for PRF) Nice, France	3 min	700 rpm	60 g
Parwani et al., 2024 [32]	NR	NR	FMS	Gracey Curettes, ultrasonic scaler	PRF clot	PC-02 (Process for PRF) Nice, France	8 min	4000 rpm	NR
Al-Rihaymee et al., 2024 [37]	Supragingival scaling, oral hygiene education	NR	FMS	Gracey curettes	PRF clot	Primefuge, model TG12 China	12 min	2700 rpm	653 g

## Data Availability

No new data were created or analyzed in this study.

## Supplementary Materials

The following supporting information can be downloaded at: https://www.mdpi.com/article/10.3390/clinpract15070127/s1, Table S1. Database search strategy. Figure S1. Funnel plot of comparison: PRF vs. Non-PRF, outcome: 1.1 Beneficial role of PRF in PPD Improvement (3 months follow-up). Figure S2. Funnel plot of comparison: PRF vs. Non-PRF, outcome: 1.2 Beneficial role of PRF in CAL Improvement (3 months follow-up). Figure S3. Funnel plot of comparison: PRF vs. Non-PRF, outcome: 1.3 Beneficial role of PRF in BoP Improvement (3 months follow-up). Figure S4. Funnel plot of comparison: PRF vs. Non-PRF, outcome: 1.4 Beneficial role of PRF in GR Improvement (3 months follow-up). Figure S5. Funnel plot of comparison: PRF vs. Non-PRF, outcome: 1.5 Beneficial role of PRF in GI Improvement (3 months follow-up). Figure S6. Funnel plot of comparison: PRF vs. Non-PRF, outcome: 1.6 Beneficial Role of PRF in PlI Improvement (3 months follow-up). Table S2. GRADE assessment. Table S3. Summary of clinical parameters from the included studies (2020–2024). Table S4. Summary of immunological parameters from the included studies (2020–2024). Table S5. PRISMA 2020 Checklist [54]. Table S6. PRISMA 2020 Checklist for Abstract [54].

## Abbreviations

The following abbreviations are used in this manuscript:

PRF	Platelet-rich fibrin
PMPR	Professional mechanical plaque removal
SRP	Scaling and root planing
NSPT	Non-surgical periodontal therapy
PPD	Probing pocket depth
CAL	Clinical attachment loss
BoP	Bleeding on probing
GR	Gingival recession
GI	Gingival index
PlI	Plaque index
IL	Interleukin
TNF-α	Tumor necrosis factor-α
VEGF	Vascular endothelial growth factor
TGF-β	Transforming growth factor-β
PDGF	Platelet-derived growth factor
GCF	Gingival crevicular fluid
