# Supplementary material for: Beneficial Effect of Platelet-Rich Fibrin as an Adjunct to Nonsurgical Therapy After Subgingival Professional Mechanical Plaque Removal for Periodontitis: A Systematic Review and Meta-Analysis
Source: Clin Pract. 2025 Jul 2;15(7):127. doi: 10.3390/clinpract15070127 (PMC12293464; doi:10.3390/clinpract15070127)
Supplement: Supplementary file 1 [file clinpract-15-00127-s001.zip › clinpract-3649528-supplementary.pdf]

Supplementary Materials

Table S1. Database search strategy.

**Database**	**Keywords**
ProQuest	("platelet-rich fibrin" OR PRF OR i-PRF OR "injectable platelet-rich fibrin") AND (periodontitis OR "periodontal disease") AND ("professional mechanical plaque removal" OR PMPR OR "subgingival instrumentation" OR "scaling and root planing" OR SRP)
PubMed	("Platelet-Rich Fibrin"[Mesh] OR "platelet-rich fibrin"[tiab] OR PRF[tiab] OR i-PRF[tiab] OR "injectable platelet-rich fibrin"[tiab]) AND ("Periodontitis"[Mesh] OR periodontitis[tiab] OR "periodontal disease"[tiab]) AND ("Plaque Removal"[Mesh] OR "professional mechanical plaque removal"[tiab] OR PMPR[tiab] OR "subgingival instrumentation"[tiab] OR "scaling and root planing"[tiab] OR SRP[tiab])
PMC	("platelet-rich fibrin"[tiab] OR PRF[tiab] OR i-PRF[tiab] OR "injectable platelet-rich fibrin"[tiab]) AND ("periodontitis"[MeSH Terms] OR periodontitis[tiab] OR "periodontal disease"[tiab]) AND ("professional mechanical plaque removal"[tiab] OR PMPR[tiab] OR "subgingival instrumentation"[tiab] OR "scaling and root planing"[tiab] OR SRP[tiab])
ScienceDirect	("Platelet-Rich Fibrin" OR "PRF") AND ("Periodontitis" OR "Periodontal Disease") AND ("Professional Mechanical Plaque Removal" OR "PMPR" OR "Subgingival Instrumentation" OR "Scaling and Root Planing" OR "SRP")
Scopus	("Platelet-Rich Fibrin" OR "PRF") AND ("Periodontitis" OR "Periodontal Disease") AND ("Professional Mechanical Plaque Removal" OR "PMPR" OR "Subgingival Instrumentation" OR "Scaling and Root Planing" OR "SRP")
EBSCO	(TX "platelet-rich fibrin" OR TX PRF OR TX i-PRF OR TX "injectable platelet-rich fibrin") AND (TX periodontitis OR TX "periodontal disease") AND (TX "professional mechanical plaque removal" OR TX PMPR OR TX "subgingival instrumentation" OR TX "scaling and root planing" OR TX SRP)

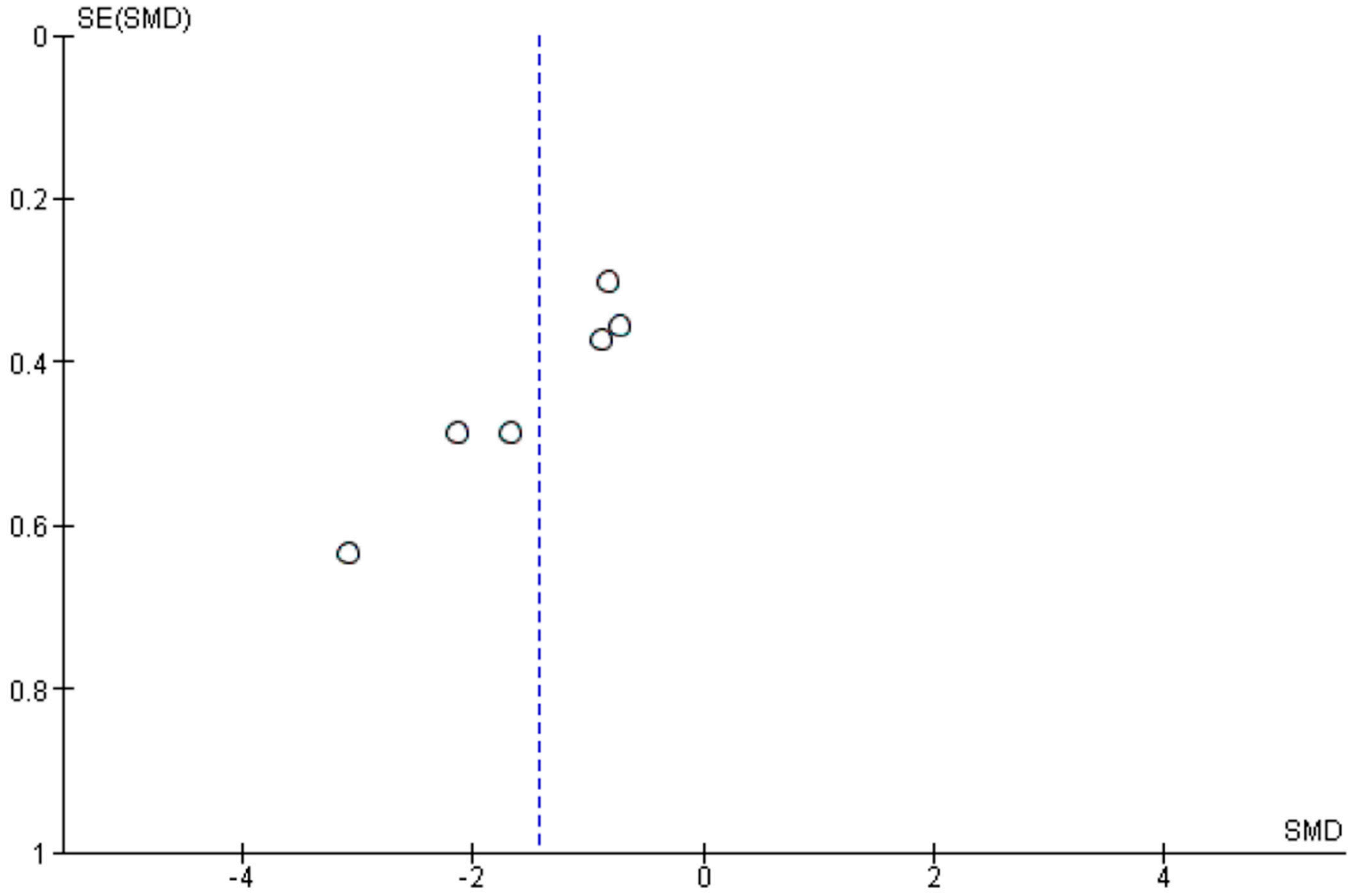

Figure S1 Funnel plot of comparison: PRF vs Non-PRF, outcome: 1.1 Beneficial Role of PRF in PPD Improvement (3 months follow up).

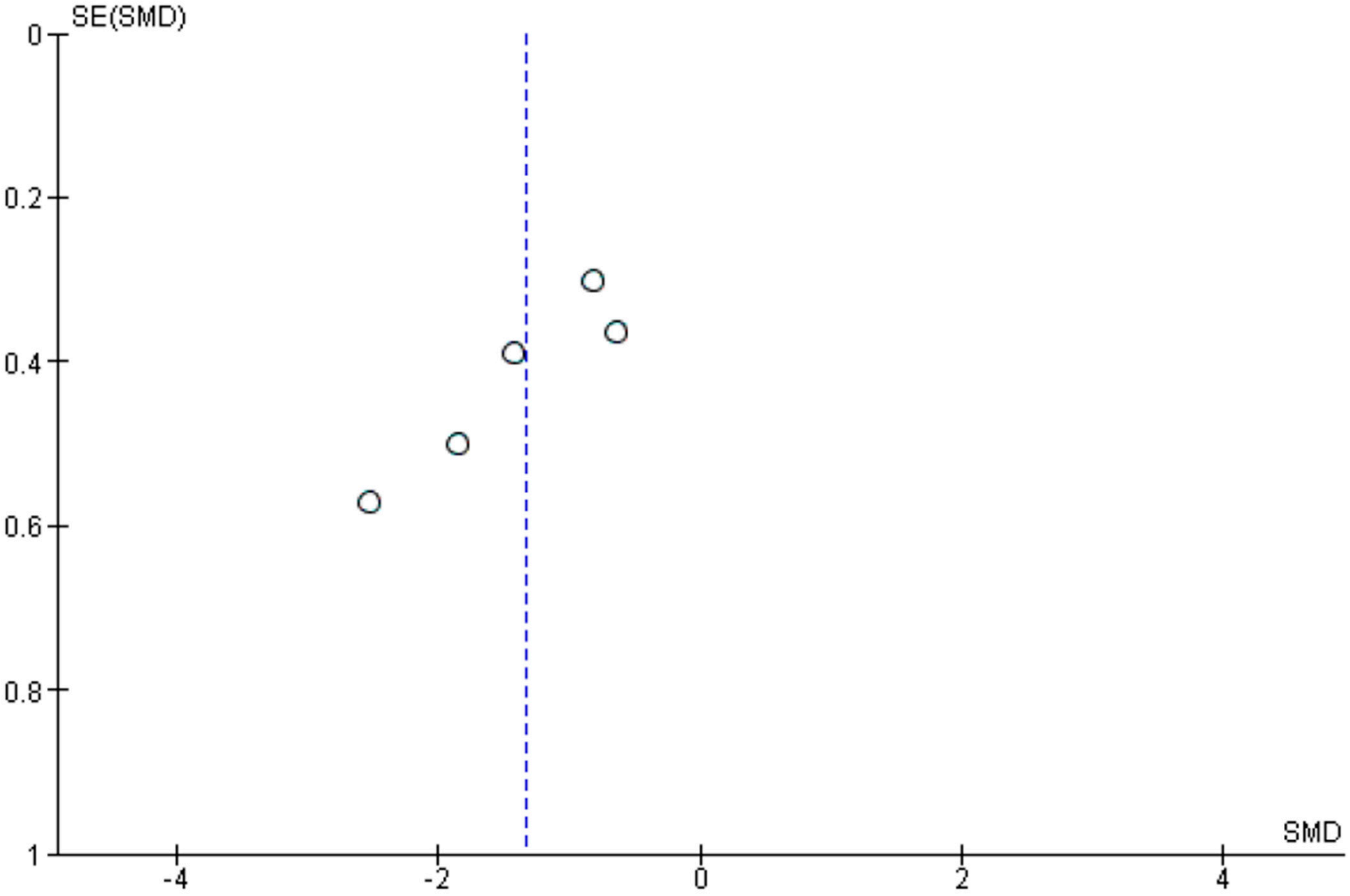

Figure S2 Funnel plot of comparison: PRF vs Non-PRF, outcome: 1.2 Beneficial Role of PRF in CAL Improvement (3 months follow up).

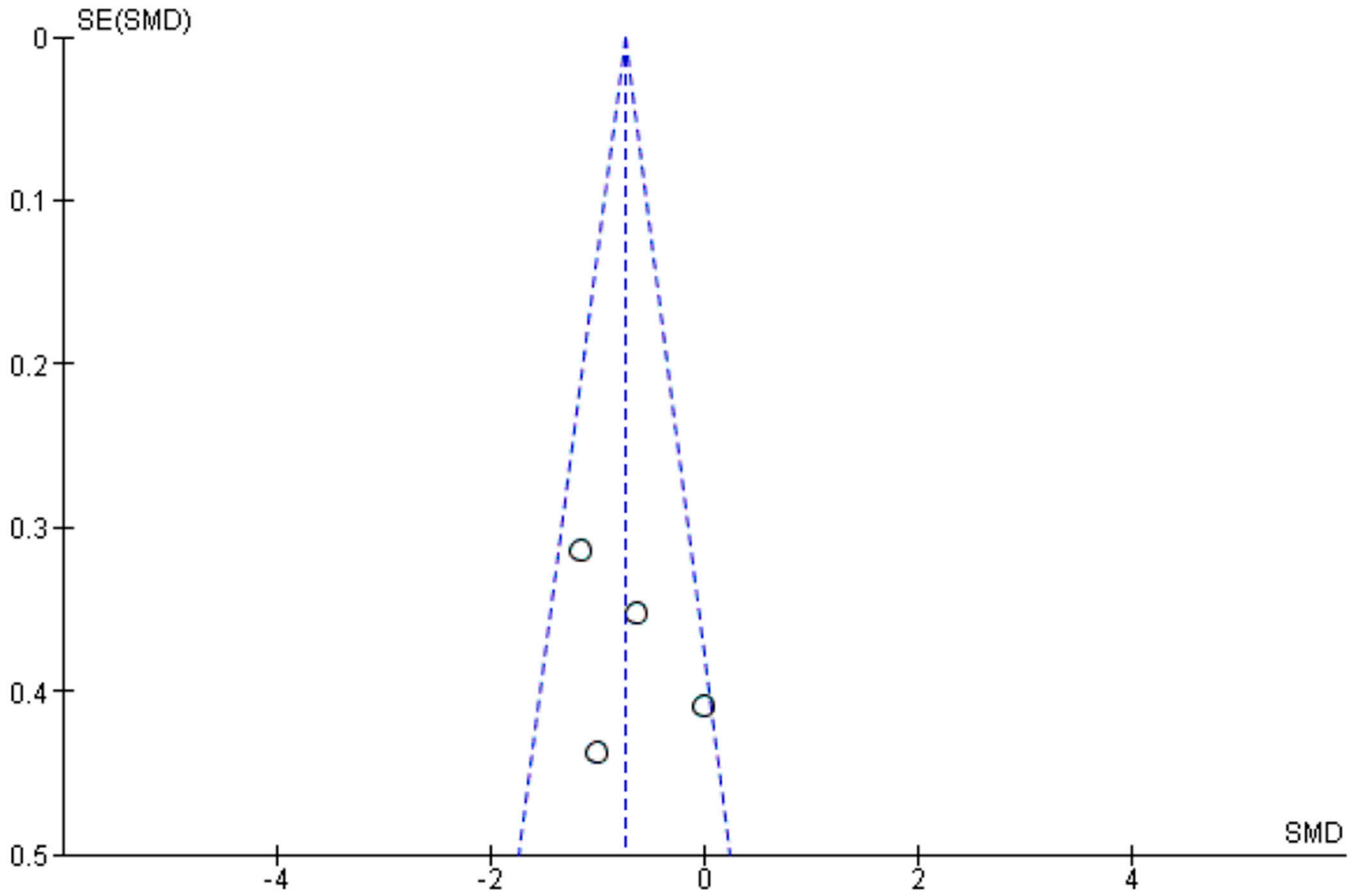

Figure S3 Funnel plot of comparison: PRF vs Non-PRF, outcome: 1.3 Beneficial Role of PRF in BoP Improvement (3 months follow up).

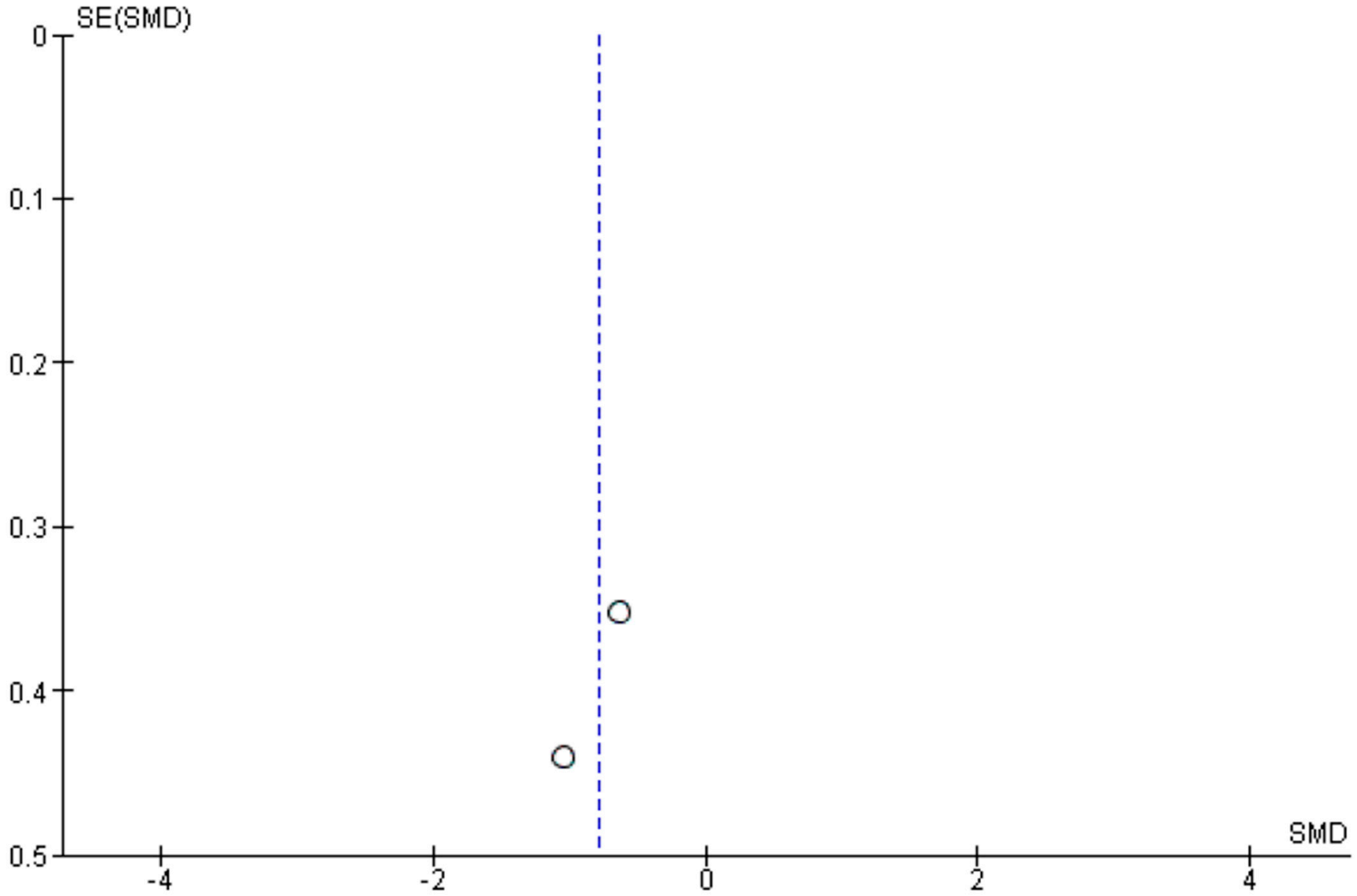

Figure S4 Funnel plot of comparison: PRF vs Non-PRF, outcome: 1.4 Beneficial Role of PRF in GR Improvement (3 months follow up).

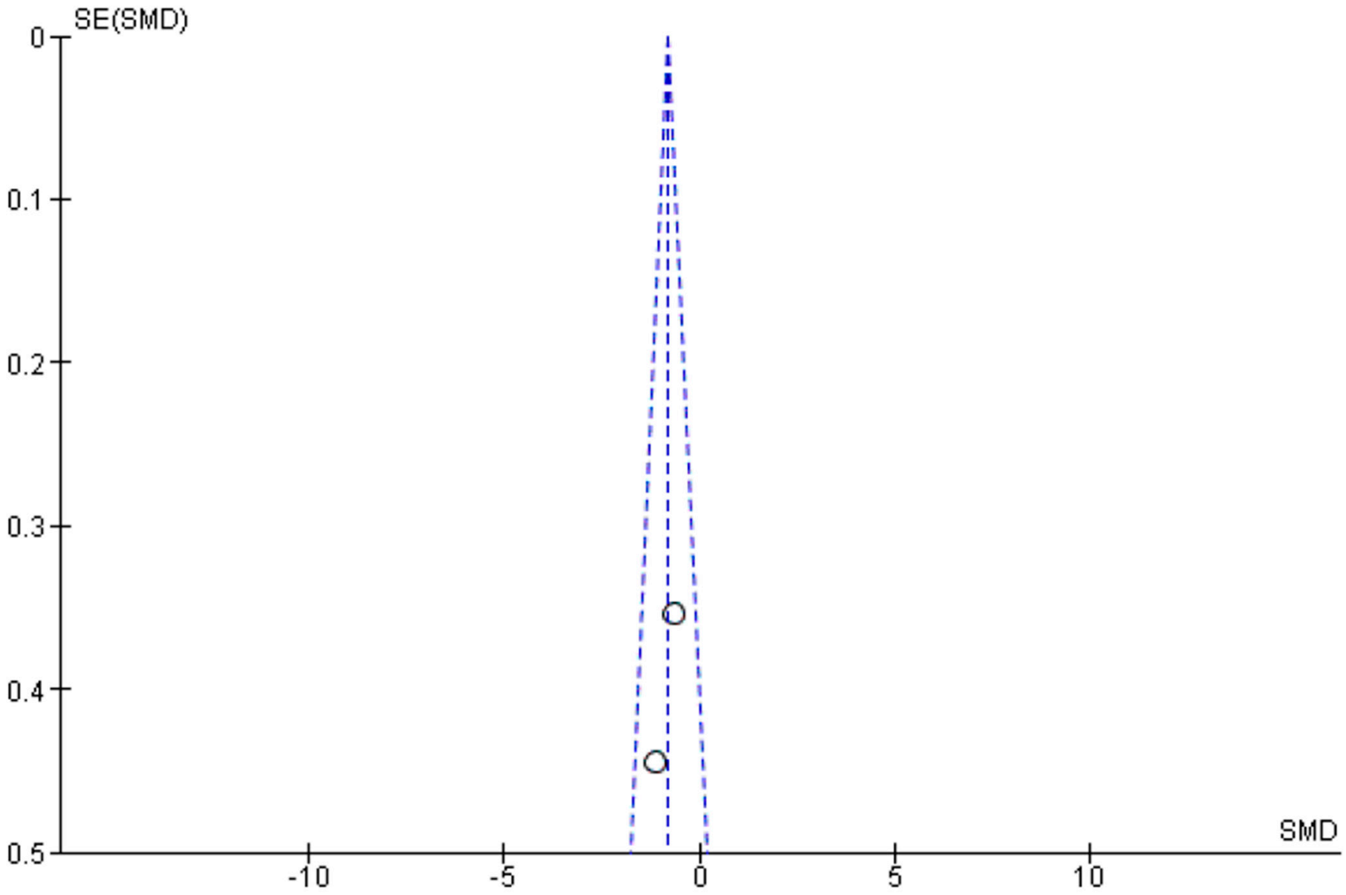

Figure S5 Funnel plot of comparison: PRF vs Non-PRF, outcome: 1.5 Beneficial Role of PRF in GI Improvement (3 months follow up).

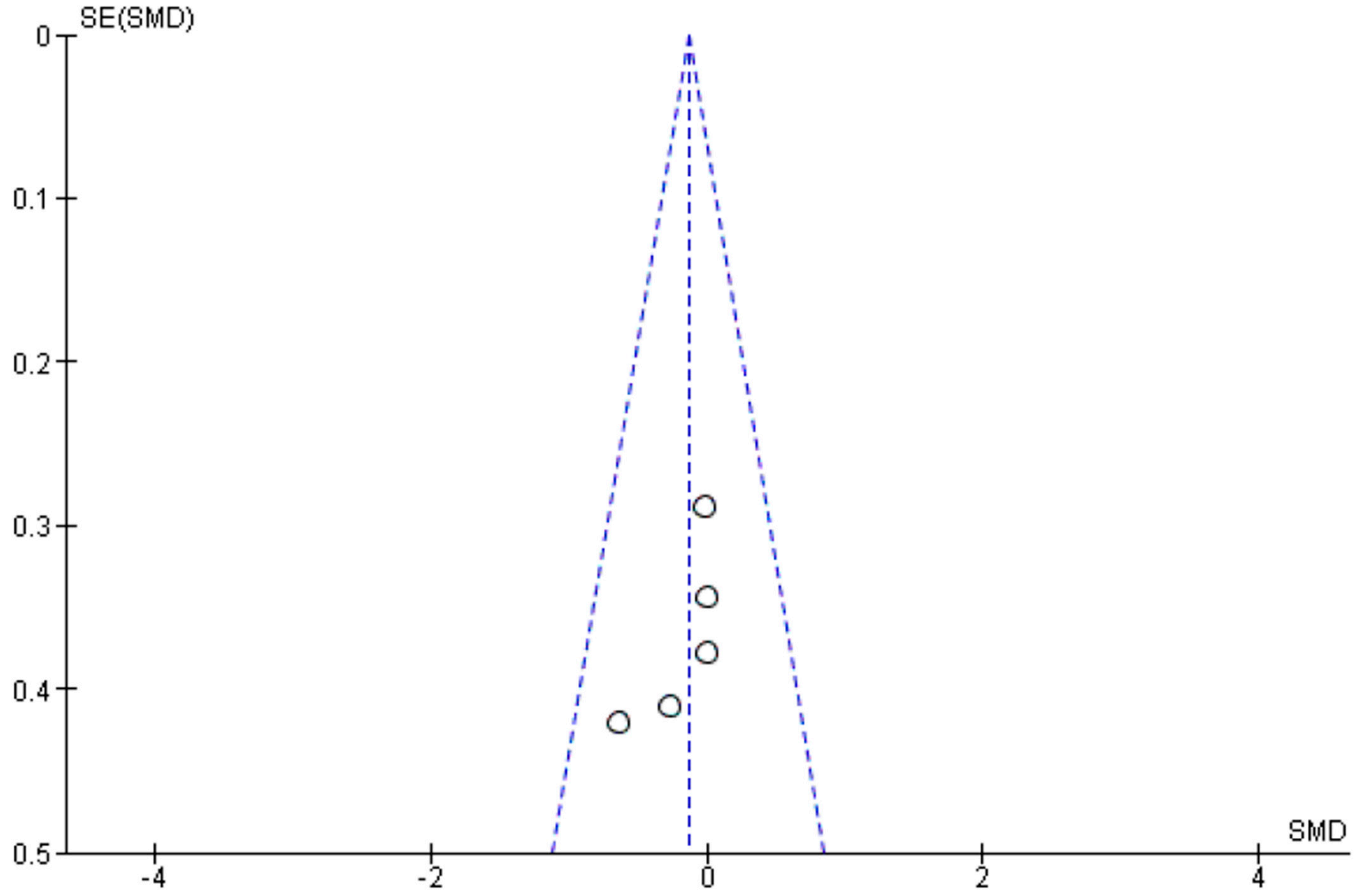

Figure S6 Funnel plot of comparison: PRF vs Non-PRF, outcome: 1.6 Beneficial Role of PRF in PlI Improvement (3 months follow up).

Table S2. GRADE assessment

Certainty assessment							№ of patients		Effect		Certainty	Importance
**№ of studies**	**Study design**	**Risk of bias**	**Inconsistency**	**Indirectness**	**Imprecision**	**Other considerations**	**PRF**	**Non-PRF**	**Relative (95% CI)**	**Absolute (95% CI)**		
**Beneficial Role of PRF in PPD Improvement (3 months follow up)**												
4	randomised trials	not serious	serious[a]	not serious	not serious	none	69	69	-	SMD **0.94 SD lower** (1.29 lower to 0.58 lower)	⨁⨁⨁◯ Moderate[a]	
**Beneficial Role of PRF in CAL Improvement (3 months follow up)**												
4	randomised trials	not serious	serious[b]	not serious	not serious	none	69	69	-	SMD **1.1 lower** (1.59 lower to 0.6 lower)	⨁⨁⨁◯ Moderate[b]	
**Beneficial Role of PRF in BoP Improvement (3 months follow up)**												
4	randomised trials	not serious	not serious	not serious	not serious	none	67	67	-	SMD **0.94 lower** (1.35 lower to 0.54 lower)	⨁⨁⨁⨁ High	
**Beneficial Role of PRF in GR Improvement (3 months follow up)**												
2	randomised trials	not serious	not serious	not serious	not serious	none	29	29	-	SMD **0.79 lower** (1.33 lower to 0.25 lower)	⨁⨁⨁⨁ High	
**Beneficial Role of PRF in GI Improvement (3 months follow up)**												
2	randomised trials	not serious	not serious	not serious	not serious	none	29	29	-	SMD **0.82 lower** (1.37 lower to 0.28 lower)	⨁⨁⨁⨁ High	
**Beneficial Role of PRF in PII Improvement (3 months follow up)**												
5	randomised trials	not serious	not serious	not serious	not serious	none	79	79	-	SMD **0.14 lower** (0.45 lower to 0.17 higher)	⨁⨁⨁⨁ High	

CI: confidence interval; **SMD:** standardized mean difference

Explanations

a. Serious unexplained inconsistency, high heterogeneity was present (I^2 = 71%)

b. Serious unexplained inconsistency, high heterogeneity was present (I^2 = 65%)

Table S3. Summary of clinical parameter from the included studies (2020–2024)

Author (Year)	Parameter	Unit	Test Group (PMPR+PRF) Follow-up period (Mean ± SD)							Control (PMPR only) Follow-up period (Mean ± SD)							
			Baseline	1 month(s)	1,5 month(s)	2 month(s)	3 month(s)	6 month(s)	*IAG* p-value*	Baseline	1 month(s)	1,5 month(s)	2 month(s)	3 month(s)	6 month(s)	*IAG* p-value*	*IEG** p-value*
Vučković dkk. (2020)	PPD	mm	3.68 ± 0.72				1.73 ± 0.64		**0.000**	3.68 ± 0.89				2.31 ± 0.73		**0.000**	**0.006**
	CAL	mm	1.97 ± 0.75				1.07 ± 0.44		**0.000**	1.81 ± 0.66				1.48 ± 0.55		**0.000**	**0.003**
	BoP	%	57.00 ± 21.00		*NR****		15.00 ± 18.00	*NR*	**0.000**	61.00 ± 17.00		*NR*		33.00 ± 12.00	*NR*	**0.000**	**0.000**
	GML	mm	1.72 ± 0.60				0.62 ± 0.49		**0.000**	1.86 ± 0.56				0.99 ± 0.57		**0.000**	**0.040**
	PlI	-	0.61 ± 0.52				0.19 ± 0.23		**0.000**	0.64 ± 0.19				0.20 ± 0.89		**0.000**	0.112
Özcan dkk. (2021)	PPD	mm	5.99 ± 1.07				3.26 ± 0.42	3.32 ± 0.40	**0.008**	5.66 ± 1.12				4.53 ± 0.95	4.50 ± 1.34	**0.021**	**0.018**
	CAL	mm	6.45 ± 1.35				3.66 ± 0.74	3.58 ± 0.79	**0.012**	5.90 ± 1.11				5.17 ± 0.84	5.31 ± 1.17	0.237	**0.012**
	BoP	%	64.05 ± 35.80		*NR*		4.57 ± 2.76	10.41 ± 5.26	**0.017**	61.27 ± 36.85		*NR*		13.66 ± 12.11	16.66 ± 11.81	**0.026**	0.180
	GR	mm	0.40 ± 0.37				0.57 ± 0.58	0.71 ± 0.86	**0.042**	0.27 ± 0.28				1.25 ± 0.68	1.79 ± 1.08	**0.005**	**0.005**
	GI	-	1.65 ± 0.44				0.22 ± 0.22	0.35 ± 0.24	**0.012**	1.59 ± 0.54				0.43 ± 0.14	0.35 ± 0.14	**0.012**	0.655
	PlI	-	1.02 ± 0.38				0.16 ± 0.18	0.43 ± 0.16	**0.005**	1.08 ± 0.44				0.27 ± 0.15	0.33 ± 0.18	**0.011**	0.339
Narendran dkk. (2022)	PPD		6.37± 0.50	*NR*		4.18 ± 1.22	3.06 ± 0.77	*NR*	**<0.001**	6.18 ± 0.66	*NR*		4.75 ± 0.86	3.93 ± 1.12	*NR*	**<0.001**	**0.002**
	CAL		6.81 ± 0.83			4.68 ± 1.08	3.44 ± 0.73		**<0.001**	6.69 ± 0.70			5.00 ± 0.97	4.13 ± 1.31		**<0.001**	**0.028**
Al-Rihaymee & Mahmood (2023)	PPD	mm	5.42 ± 0.64	2.89 ± 0.34			2.10 ± 0.28		**0.001**	4.92 ± 0.73	3.71 ± 0.61			3.03 ± 0.53		**0.001**	**0.000**
	RAL	mm	7.92 ± 0.73	5.31 ± 0.64	*NR*		4.60 ± 0.68	*NR*	**0.001**	7.35 ± 1.08	6.07 ± 1.03	*NR*		5.51 ± 0.86	*NR*	**0.001**	**0.006**
	BoP	%	35.70 ± 49.70	0.00 ± 0.00			0.00 ± 0.00		**0.025**	35.70 ± 49.70	0.00 ± 0.00			0.00 ± 0.00		**0.025**	1.000
	PlI	-	1.71 ± 0.82	0.36 ± 0.49			0.14 ± 0.36		**0.001**	0.93 ± 0.73	0.36 ± 0.49			0.14 ± 0.36		**0.001**	1.000
Cin dkk. (2023)	PPD	mm	7.76 ± 0.75	5.76 ± 0.97			5.47 ± 1.07	5.41 ± 1.08	**0.000**	7.71 ± 0.68	6.47 ± 0.87			6.18 ± 0.80	6.06 ± 1.03	**0.000**	**0.048**
	CAL	mm	8.24 ± 0.83	6.59 ± 1.06			6.48 ± 0.99	6.42 ± 0.95	**0.000**	8.29 ± 0.58	7.82 ± 0.88			7.76 ± 0.75	7.71 ± 0.84	**0.001**	**0.004**
	BoP	%	74.12 ± 11.61	11.29 ± 4.16	*NR*		11.88 ± 5.24	15.94 ± 6.86	**0.000**	76.06 ± 13.47	14.00 ± 5.63	*NR*		15.35 ± 5.54	19.00 ± 7.15	**0.000**	0.213
	GR	mm	0.47 ± 0.51	0.76 ± 0.56			1.18 ± 0.58	1.48 ± 0.52	**0.000**	0.59 ± 0.61	1.29 ± 0.58			1.53 ± 0.51	1.82 ± 0.56	**0.000**	**0.035**
	GI	-	1.69 ± 0.19	0.34 ± 0.11			0.36 ± 0.09	0.57 ± 0.14	**0.000**	1.67 ± 0.16	0.36 ± 0.10			0.42 ± 0.09	0.60 ± 0.14	**0.000**	0.512
	PlI	-	1.32 ± 0.23	0.30 ± 0.16			0.36 ± 0.12	0.55 ± 0.10	**0.000**	1.23 ± 0.28	0.33 ± 0.10			0.36 ± 0.05	0.54 ± 0.15	**0.000**	0.839
Parwani dkk. (2024)	PPD	mm	5.31 ± 0.63		2.62 ± 0.65				**0.001**	5.15 ± 0.56		3.85 ± 0.80				**0.001**	**0.001**
	CAL	mm	5.54 ± 0.52		2.69 ± 0.63				**0.001**	5.38 ± 0.51		4.15 ± 0.69				**0.001**	**0.001**
	GR	mm	0.77 ± 0.53	*NR*	0.46 ± 0.62		*NR*		0.440	1.04 ± 0.78	*NR*	0.81 ± 0.72		*NR*		0.190	0.210
	GI	-	2.62 ± 0.65		1.08 ± 0.49				**0.001**	2.77 ± 0.44		1.77 ± 0.44				**0.001**	**0.001**
	PlI	-	2.15 ± 0.38		0.54 ± 0.52				**0.001**	2.38 ± 0.65		1.38 ± 0.65				**0.001**	**0.001**
Al-Rihaymee dkk. (2024)	PPD	mm	5.41 ± 0.70	2.83 ± 0.32			2.10 ± 0.31		**<0.001**	4.60 ± 0.94	3.70 ± 0.39			3.33 ± 0.45		**<0.001**	**<0.000**
	CAL	mm	6.80 ± 0.70	4.20 ± 0.45	*NR*		3.54 ± 0.39	*NR*	**<0.001**	6.13 ± 0.97	5.10 ± 0.48	*NR*	*NR*	4.70 ± 0.49	*NR*	**<0.001**	**<0.000**
	BoP	%	58.31 ± 16.65	6.90 ± 3.12			8.31 ± 5.30		**<0.001**	58.32 ± 16.70	6.92 ± 3.11			8.31 ± 5.28		**<0.001**	1.000
	PlI	-	1.75 ± 0.90	0.42 ± 0.21			0.16 ± 0.03		**<0.001**	1.90 ± 0.79	0.42 ± 0.05			0.17 ± 0.04		**<0.001**	1.000

p-value < 0.05, significant, bold; * intragroup p-value (IAG) recorded from baseline to latest follow-up period; ** intergroup p-value (IEG) comparison of test and control groups at latest follow-up period; *** NR: not reported

Table S4. Summary of immunological parameter from the included studies (2020–2024)

Author (Year)	Parameter	Unit	Test Group (PMPR+PRF) Follow-up period (Mean ± SD)							Kelompok Kontrol Periode *Follow-up* (Rerata ± SD)							*nilai-p IEG***
			Baseline	3 day(s)	7 day(s)	14 day(s)	1 month(s)	3 month(s)	*IAG* p-value*	Baseline	3 day(s)	7 day(s)	14 day(s)	1 month(s)	3 month(s)	nilai-p *IAG**	
Özcan dkk. (2021)	*TGF-β*	pg/situs	15.20 ± 4.31	11.93 ± 2.68	12.54 ± 3.66	17.19 ± 11.66	*NR****		0.937	16.85 ± 9.69	6.34 ± 1.67	6.35 ± 3.44	7.51 ± 2.85	*NR****		**0.003**	**0.003**
	Kolagen tipe-I	ng/situs	0.74 ± 0.46	1.27 ± 1.05	1.35 ± 0.76	0.97 ± 0.53			0.195	0.83 ± 0.79	0.76 ± 0.20	0.84 ± 0.24	0.57 ± 0.19			0.155	**0.002**
Al-Rihaymee & Mahmood (2023)	Periostin	(ng/µl)	26.35 ± 6.53		NR		48.83 ± 9.30	98.90 ± 24.94	**0.000**	26.70 ± 6.51		NR		42.65 ± 7.03	49.29 ± 15.14	**0.000**	**0.000**
Cin dkk. (2023)	*VEGF*	ng/L	0.40 ± 0.03	NR	0.35 ± 0.04	0.48 ± 0.05	*NR*		**0.000**	0.41 ± 0.04	NR	0.36 ± 0.04	0.32 ± 0.04	*NR*		**0.000**	**0.000**
	TNF-α	ng/L	0.09 ± 0.01		0.06 ± 0.01	0.04 ± 0.01			**0.000**	0.09 ± 0.02		0.08 ± 0.01	0.07 ± 0.01			**0.000**	**0.000**
	IL-10	pg/ml	1.82 ± 0.33		2.24 ± 0.41	2.68 ± 0.45			**0.000**	1.87 ± 0.21		2.21 ± 0.28	1.83 ± 0.37			**0.003**	**0.001**
Al-Rihaymee dkk. (2024)	*PDGF-BB*	(ng/µl)	712.30 ± 216.80		NR		1436.80 ± 402.89	2669.80 ± 689.70	**<0.001**	576.78 ± 124.00		NR		686.08 ± 99.93	1191.42 ± 333.33	**<0.001**	**0.001**

*p-value < 0.05, significant, bold; * intragroup p-value (IAG) recorded from baseline to latest follow-up period; ** intergroup p-value (IEG) comparison of test and control groups at latest follow-up period; *** NR: not reported*

Table S5. PRISMA 2020 Checklist

Section and Topic	Item #	Checklist item	Location where item is reported
**TITLE**			
Title	1	Identify the report as a systematic review.	Title, page 1
**ABSTRACT**			
Abstract	2	See the PRISMA 2020 for Abstracts checklist.	*see table below
**INTRODUCTION**			
Rationale	3	Describe the rationale for the review in the context of existing knowledge.	Section 1, page 1–2
Objectives	4	Provide an explicit statement of the objective(s) or question(s) the review addresses.	Section 1, page 3
**METHODS**			
Eligibility criteria	5	Specify the inclusion and exclusion criteria for the review and how studies were grouped for the syntheses.	Sections 2.3 and 2.4, page 4
Information sources	6	Specify all databases, registers, websites, organisations, reference lists and other sources searched or consulted to identify studies. Specify the date when each source was last searched or consulted.	Section 2.2, page 3–4
Search strategy	7	Present the full search strategies for all databases, registers and websites, including any filters and limits used.	Section 2.2, page 3
Selection process	8	Specify the methods used to decide whether a study met the inclusion criteria of the review, including how many reviewers screened each record and each report retrieved, whether they worked independently, and if applicable, details of automation tools used in the process.	Section 2.5, page 4–5
Data collection process	9	Specify the methods used to collect data from reports, including how many reviewers collected data from each report, whether they worked independently, any processes for obtaining or confirming data from study investigators, and if applicable, details of automation tools used in the process.	Section 2.5, page 4–5
Data items	10a	List and define all outcomes for which data were sought. Specify whether all results that were compatible with each outcome domain in each study were sought (e.g. for all measures, time points, analyses), and if not, the methods used to decide which results to collect.	Section 2.2, page 3–4<br>Section 2.5, page 4–5
	10b	List and define all other variables for which data were sought (e.g. participant and intervention characteristics, funding sources). Describe any assumptions made about any missing or unclear information.	Section 2.5, page 4–5
Study risk of bias assessment	11	Specify the methods used to assess risk of bias in the included studies, including details of the tool(s) used, how many reviewers assessed each study and whether they worked independently, and if applicable, details of automation tools used in the process.	Section 2.6, page 5
Effect measures	12	Specify for each outcome the effect measure(s) (e.g. risk ratio, mean difference) used in the synthesis or presentation of results.	Section 2.7, page 5
Synthesis methods	13a	Describe the processes used to decide which studies were eligible for each synthesis (e.g. tabulating the study intervention characteristics and comparing against the planned groups for each synthesis (item #5)).	Section 2.7, page 5
	13b	Describe any methods required to prepare the data for presentation or synthesis, such as handling of missing summary statistics, or data conversions.	Section 2.7, page 5

Section and Topic	Item #	Checklist item	Location where item is reported
	13c	Describe any methods used to tabulate or visually display results of individual studies and syntheses.	Section 2.7, page 5
	13d	Describe any methods used to synthesize results and provide a rationale for the choice(s). If meta-analysis was performed, describe the model(s), method(s) to identify the presence and extent of statistical heterogeneity, and software package(s) used.	Section 2.7, page 5
	13e	Describe any methods used to explore possible causes of heterogeneity among study results (e.g. subgroup analysis, meta-regression).	Section 2.7, page 5
	13f	Describe any sensitivity analyses conducted to assess robustness of the synthesized results.	Section 2.7, page 5
Reporting bias assessment	14	Describe any methods used to assess risk of bias due to missing results in a synthesis (arising from reporting biases).	Section 2.6, page 5
Certainty assessment	15	Describe any methods used to assess certainty (or confidence) in the body of evidence for an outcome.	Section 2.7, page 5
**RESULTS**			
Study selection	16a	Describe the results of the search and selection process, from the number of records identified in the search to the number of studies included in the review, ideally using a flow diagram.	Section 3.1, page 5–8
	16b	Cite studies that might appear to meet the inclusion criteria, but which were excluded, and explain why they were excluded.	Section 3.1, page 5–8
Study characteristics	17	Cite each included study and present its characteristics.	Section 3.1, page 5–8
Risk of bias in studies	18	Present assessments of risk of bias for each included study.	Section 3.2, page 8–9
Results of individual studies	19	For all outcomes, present, for each study: (a) summary statistics for each group (where appropriate) and (b) an effect estimate and its precision (e.g. confidence/credible interval), ideally using structured tables or plots.	Section 3.4, page 9
Results of syntheses	20a	For each synthesis, briefly summarise the characteristics and risk of bias among contributing studies.	Sections 3.4 and 3.5, page 9
	20b	Present results of all statistical syntheses conducted. If meta-analysis was done, present for each the summary estimate and its precision (e.g. confidence/credible interval) and measures of statistical heterogeneity. If comparing groups, describe the direction of the effect.	Section 3.5.1 – 3.5.6, page 9–13
	20c	Present results of all investigations of possible causes of heterogeneity among study results.	Section 3.5.1 and 3.5.2, page 10–11
	20d	Present results of all sensitivity analyses conducted to assess the robustness of the synthesized results.	Section 3.5.1 and 3.5.2, page 10–11
Reporting biases	21	Present assessments of risk of bias due to missing results (arising from reporting biases) for each synthesis assessed.	Section 3.2 and 3.3, page 8–9
Certainty of evidence	22	Present assessments of certainty (or confidence) in the body of evidence for each outcome assessed.	Section 3.3, page 9
**DISCUSSION**			
Discussion	23a	Provide a general interpretation of the results in the context of other evidence.	Sections 4.1 – 4.3, page 14–16

Section and Topic	Item #	Checklist item	Location where item is reported
	23b	Discuss any limitations of the evidence included in the review.	Section 4.4, page 16–17
	23c	Discuss any limitations of the review processes used.	Section 4.4, page 16–17
	23d	Discuss implications of the results for practice, policy, and future research.	Section 4.4, page 16–17
**OTHER INFORMATION**			
Registration and protocol	24a	Provide registration information for the review, including register name and registration number, or state that the review was not registered.	Section 2.1, page 3
	24b	Indicate where the review protocol can be accessed, or state that a protocol was not prepared.	Section 2.7, page 5
	24c	Describe and explain any amendments to information provided at registration or in the protocol.	Appendix, page 18
Support	25	Describe sources of financial or non-financial support for the review, and the role of the funders in the review.	Funding, page 18
Competing interests	26	Declare any competing interests of review authors.	Conflict of Interest, page 18
Availability of data, code and other materials	27	Report which of the following are publicly available and where they can be found: template data collection forms; data extracted from included studies; data used for all analyses; analytic code; any other materials used in the review.	Supplementary, page 17

From: Page MJ, McKenzie JE, Bossuyt PM, Boutron I, Hoffmann TC, Mulrow CD, et al. The PRISMA 2020 statement: an updated guideline for reporting systematic reviews. BMJ 2021;372:n71. doi: 10.1136/bmj.n71. This work is licensed under CC BY 4.0. To view a copy of this license, visit https://creativecommons.org/licenses/by/4.0/

Table S6. PRISMA 2020 Checklist for Abstract

Section and Topic	Item #	Checklist item	Reported (Yes/No)
**TITLE**			
Title	1	Identify the report as a systematic review.	Yes
**BACKGROUND**			
Objectives	2	Provide an explicit statement of the main objective(s) or question(s) the review addresses.	Yes
**METHODS**			
Eligibility criteria	3	Specify the inclusion and exclusion criteria for the review.	Yes
Information sources	4	Specify the information sources (e.g. databases, registers) used to identify studies and the date when each was last searched.	Yes
Risk of bias	5	Specify the methods used to assess risk of bias in the included studies.	Yes
Synthesis of results	6	Specify the methods used to present and synthesise results.	Yes
**RESULTS**			
Included studies	7	Give the total number of included studies and participants and summarise relevant characteristics of studies.	Yes
Synthesis of results	8	Present results for main outcomes, preferably indicating the number of included studies and participants for each. If meta-analysis was done, report the summary estimate and confidence/credible interval. If comparing groups, indicate the direction of the effect (i.e. which group is favoured).	Yes
**DISCUSSION**			
Limitations of evidence	9	Provide a brief summary of the limitations of the evidence included in the review (e.g. study risk of bias, inconsistency and imprecision).	Yes
Interpretation	10	Provide a general interpretation of the results and important implications.	Yes
**OTHER**			
Funding	11	Specify the primary source of funding for the review.	Yes
Registration	12	Provide the register name and registration number.	Yes

From: Page MJ, McKenzie JE, Bossuyt PM, Boutron I, Hoffmann TC, Mulrow CD, et al. The PRISMA 2020 statement: an updated guideline for reporting systematic reviews. BMJ 2021;372:n71. doi: 10.1136/bmj.n71. This work is licensed under CC BY 4.0. To view a copy of this license, visit https://creativecommons.org/licenses/by/4.0/
